# Genomic copy number variation in *Mus musculus*

**DOI:** 10.1186/s12864-015-1713-z

**Published:** 2015-07-04

**Authors:** M Elizabeth O Locke, Maja Milojevic, Susan T Eitutis, Nisha Patel, Andrea E Wishart, Mark Daley, Kathleen A Hill

**Affiliations:** Department of Computer Science, The University of Western Ontario, London, ON N6A 5B7 Canada; Department of Biology, The University of Western Ontario, Biological and Geological Sciences Building 1151 Richmond St. N, London, ON N6A 5B7 Canada

**Keywords:** Copy number variation, Single nucleotide polymorphism, Genotyping, Genomic, Genetic distance, Genetic background, *Mus musculus*, Mouse diversity genotyping array, Genetic variation, Gene enrichment

## Abstract

**Background:**

Copy number variation is an important dimension of genetic diversity and has implications in development and disease. As an important model organism, the mouse is a prime candidate for copy number variant (CNV) characterization, but this has yet to be completed for a large sample size. Here we report CNV analysis of publicly available, high-density microarray data files for 351 mouse tail samples, including 290 mice that had not been characterized for CNVs previously.

**Results:**

We found 9634 putative autosomal CNVs across the samples affecting 6.87 % of the mouse reference genome. We find significant differences in the degree of CNV uniqueness (single sample occurrence) and the nature of CNV-gene overlap between wild-caught mice and classical laboratory strains. CNV-gene overlap was associated with lipid metabolism, pheromone response and olfaction compared to immunity, carbohydrate metabolism and amino-acid metabolism for wild-caught mice and classical laboratory strains, respectively. Using two subspecies of wild-caught *Mus musculus*, we identified putative CNVs unique to those subspecies and show this diversity is better captured by wild-derived laboratory strains than by the classical laboratory strains. A total of 9 genic copy number variable regions (CNVRs) were selected for experimental confirmation by droplet digital PCR (ddPCR).

**Conclusion:**

The analysis we present is a comprehensive, genome-wide analysis of CNVs in *Mus musculus*, which increases the number of known variants in the species and will accelerate the identification of novel variants in future studies.

**Electronic supplementary material:**

The online version of this article (doi:10.1186/s12864-015-1713-z) contains supplementary material, which is available to authorized users.

## Background

While current methods uncover ever greater quantities of copy number variants (CNVs) relevant to complex phenotypes and using increasingly sophisticated sample designs, challenges persist in accurate and sensitive detection and confirmation of CNV calls. In humans, the study of CNV associations with complex phenotypes is in high demand with a rich diversity of cost-effective methods [1, 2]; challenges in experimental design lie in the limited availability of relevant tissue samples. The opposite situation exists for CNV analyses in mice, where biological samples are not limiting, but high-throughput technologies lack resolution and variety. Also, bioinformatic resources like genomic annotations are more limited and software are not always useable out of the box with mouse data.

Studies of CNVs in mice have relied on two main approaches. The first, array comparative genomic hybridization (aCGH), reports relative copy number to a reference (C57BL/6 J) [3–10]. These studies established the extent of copy number variation in the mouse and impact of CNVs on differential expression and phenotypic variation. Taken together, they have characterized around 70 strains of mice, as well as wild-caught mice. The second approach is next-generation sequencing (NGS) [11–16], which allows for much higher resolution and accuracy, as well as the ability to further characterize the mechanisms and structural variants (SVs) resulting in CNV events. NGS also has the sensitivity to detect SVs, that are not detectable by array-based methods, such as inversions, novel insertions, small insertions, small deletions and complex indel mutations. While NGS is the method of choice for modern structural variant analysis, it still remains prohibitively expensive for many projects, but has been completed for 18 strains [11, 13, 17].

For studying the human genome, high-density single nucleotide polymorphism (SNP) microarrays have become a common platform for CNV analysis and were used as part of the HapMap project [18]. Large-scale studies have also used SNP microarrays for dog, swine and cow [19–21]. In contrast to aCGH, originally the SNP microarray’s primary purpose was for genotyping, with probe sets designed to distinguish the genotype at sites of known polymorphism. The arrays may also include probe sets designed for sites where there is no known SNP variation, called copy number (CN) probes or invariant genomic probes (IGPs). Taken together, the SNP and IGP probe sets can be used with various available algorithms [1, 2] to identify putative CNV calls.

The Affymetrix^®^ Mouse Diversity Genotyping Array (MDGA) is the most dense SNP array currently available and also includes IGPs relevant to CNV analysis [22]. The MDGA has been used to characterize and map the subspecific origin (from the three main *Mus musculus* subspecies; *domesticus, musculus and castaneus*) and haplotype diversity of SNPs for 198 samples including wild-caught mice, wild-derived laboratory strains and classical laboratory strains [23]. Many of the wild-derived strains, thought to be faithful representatives of related wild-caught mice, showed introgression. Classical laboratory strains were derived mostly from *M. m. domesticus*, with the other main contributor being *M. m. molossinus*. SNP probe sets on the array were identified where unknown genetic variation affected probe set performance, termed variable intensity oligonucleotides (VINOs). VINOs may represent off-target variation in the genome near the SNP queried by a probe set and show a consistent, low-intensity cluster during genotyping. Didion *et al.* extended the work using 351 mouse samples and showed that inclusion of VINOs in analysis reduces ascertainment bias as well as improves accuracy of the results [24]. Using the MDGA, introgression was also shown across subspecies boundaries in natural populations of *M. m. domesticus* and *M. m. musculus* [25]. This introgression was shown to affect more than 10 % of the genome, and showed evidence of positive selection. The MDGA has also been used to characterize copy number alterations (CNAs) in tumourigenesis, where incremental accumulation of CNAs was seen during tumour development [26]. However, the MDGA has yet to be applied to a large population of mice for CNV characterization.

Here, we report CNV analysis of 351 mice using the MDGA and analyzed with PennCNV software, representing 290 strains that have not been studied for CNVs previously. We compare these putative CNVs to those found by both NGS and aCGH studies, identify and analyze recurrent CNV regions and characterize the genes and pathways affected by putative CNV regions. CN confirmation in three commonly used classical laboratory strains was performed using droplet digital PCR (ddPCR). Nine genic copy number variable regions (CNVRs) that differ in copy number between classical inbred strains were selected for CNV confirmation in five C57BL/6 J, five CBA/CaJ and four DBA/2 J mice. Furthermore, we compare the CNV distance to the SNP distance between the Mouse Genomics Institute (MGI) priority strains and discuss the MDGA and its use in CNV studies.

## Results and discussion

### CNVs detected

Using ~4.8 million probes, filtered from the Affymetrix^®^ Mouse Diversity Genotyping Array (MDGA), we analyzed CNV content in a diverse set of 351 publically available array intensity CEL files [27]. Probe sets were filtered to reduce possible sources of noise and false positives in CNV detection (see Additional file 1, Figure S1). SNP and IGP probe sets targeting 925,378 unique loci (see Additional file 2), have an inter-probe-set median distance of 319 bp. CNVs were identified using PennCNV software separately for autosomes and the X chromosome. CNVs were filtered to include calls between 500 bp to 1 Mb, having a minimum probe density of approximately one probe per 7700 bp. For samples to be included in the main analysis, their data must have passed two quality control criteria for the autosomes; small log-R ratio standard deviation (LRR_SD < 0.35) and low drift in B-allele frequency (BAF drift < 0.01). All data are provided as a resource to researchers in Additional files 3, 4, 5 and 6.

For 334 samples passing quality control criteria, a total of 9634 CNVs were identified on the autosomes, with an average of 28.84 calls per sample (Table 1). On the X chromosome, 1218 CNVs were found (see Additional file 1: Tables S1 and S2), with an average of 3.65 calls per sample. Calls across all samples affect 6.87 % (169.9 Mb) of the autosomal genome or 8.15 % (215.2 Mb) when including calls on the X chromosome. Studies have found between 1.2 % [11] and 10.7 % [8] of the reference genome affected by SVs and CNVs respectively. The percent of the genome affected was higher for wild-derived mouse samples at 3.4 % [11], and in a study including wild-caught samples at 10.7 % [8]. These values are all affected by the sample size, capture technology and diversity of samples, which differs between studies. The amount of the mouse genome affected by CNVs is greater than that reported for dog (1.08 %) [19], cattle (1.61 %–4.60 %) [21, 28] and swine (4.23 %) [20] but is similar to that reported for humans (3.7 %, 7.6 %, 12 %) [29–31].

**Table 1:** Number of CNV calls on the autosomes by mouse classification and copy number state

Mouse classification	Number of samples	CNV calls		Copy number state^a^						Del/amp^b^
				0		1		3+		
All	334	9634	(28.84)	1995	(5.97)	3661	(10.96)	3978	(11.91)	1.42
Classical	114	2824	(24.77)	424	(3.72)	867	(7.61)	1533	(13.45)	0.84
Wild Derived	52	2611	(50.21)	1214	(23.35)	594	(11.42)	803	(15.44)	2.25
Wild Caught	19	969	(51.0)	231	(12.15)	491	(25.84)	247	(13.0)	2.92
C57BL/6 J	8	90	(11.52)	0	(0.0)	38	(4.75)	52	(6.5)	0.73
C57BL/6NJ	6	46	(7.67)	5	(0.83)	23	(3.83)	18	(3.0)	1.56

Values in parentheses are normalized by sample count

^a^ Copy number 0 is a full deletion, or no copies, then 1 copy, then 3 or more copies respectively

^b^ Deletion/Amplification is the total number of deletions (0 and 1 copy-state call counts) divided by the number of amplifications (3+ copy-state call counts)

Strains classified as classical laboratory strains have a mean of 0.065 % (1.6 Mb) of the autosomes affected by CNVs, 0.065 % (1.7 Mb) when the X chromosome was included. The mean autosome and genome percentage affected for the wild-derived laboratory strains (0.15 % or 3.6 Mb and 0.14 % or 3.8 Mb, respectively) and wild-caught mice (0.14 % or 3.5 Mb and 0.14 % or 3.8 Mb, respectively) were significantly different than the classical laboratory strains (*P* < 0.01, Mann–Whitney test).

**Fig. 1:**
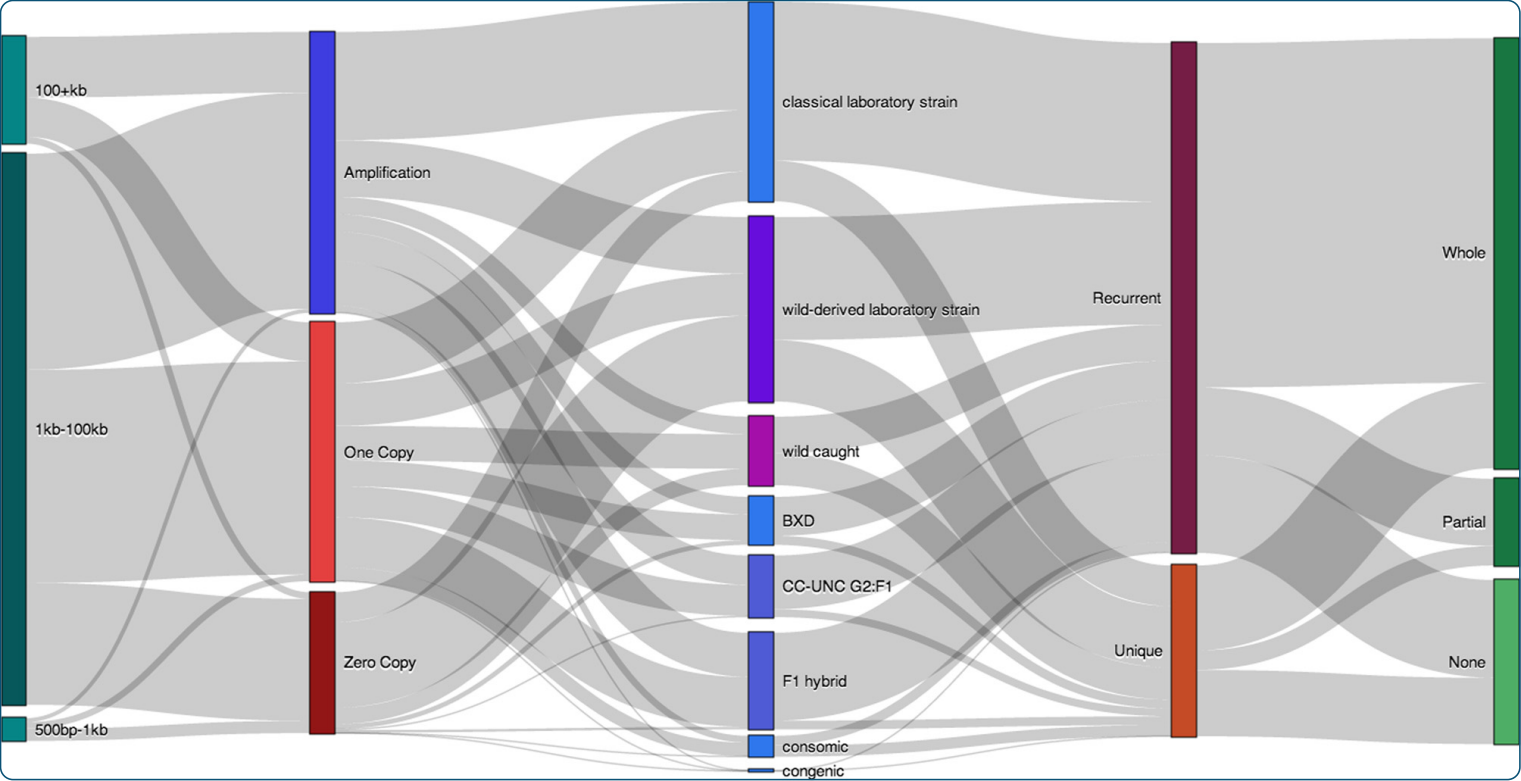
CNV call summary. Sankey diagram depicting CNV calls on the autosomes classified into unique categories stacked vertically for length, type, mouse strain type, uniqueness and gene content from left to right. Flows between vertical categories (in grey) are proportional to the number of calls sharing both horizontally neighboring classifications. For example, almost half of the “100 + kb” classified CNV calls are also “Amplifications”

The CNVs on the autosomes have an average length of 54,037 bp, with a median length of 26,340 bp. The majority (81 %) of CNV calls are between 1 kb and 100 kb (Fig. 1). Amplifications are significantly larger than deletions (*P* < 2.2 × 10^−16^, Mann–Whitney test), where amplifications have a median length of 36,708 bp compared to deletions at 20,091 bp. Copy-state-zero deletions are significantly smaller than copy-state-one deletions (*P* < 2.2 × 10^−16^, Mann–Whitney test), where copy-state-zero deletions have a median length of 13,766 bp compared to copy-state-one deletions at 26,980 bp. Deletions outnumber amplifications by a ratio of 1.42:1 on the autosomes (Table 1), which is consistent with previous studies [14].

### Concordance with previous studies

We performed strain-matched comparisons of our calls with those found with next generation sequencing methods by the Sanger mouse genome project ([17] Release SV 1302) with a relatively stringent overlap criterion of 20 % reciprocal overlap, as well as a more relaxed criterion of single base pair overlap and found concordance ranging from 0 % - 76.2 %, with a median concordance of 33.3 % (Additional file 1: Table S3). The lowest concordance was found in the C57BL/6NJ mice, the highest in the NZO/HiLtJ (76.2 %). Low concordance in C57BL/6NJ is not unexpected, as we found few CNV calls in this strain (ranging from 4–12 calls), as well as few reported variants in the Sanger data (212). Calls that overlapped known SV from the Sanger data tended to be longer than those not found (median length 31036 and 15015 respectively, Mann Whitney *U* test *p* = 4.17 × 10^−6^). The copy-number state of the call also affected how often overlap was observed, with copy-state 1 least often being found to overlap any calls in the Sanger data, and copy-state 0 most often found to overlap calls (Additional file 1: Table S4). The percentage of calls which overlapped Sanger data, that were found across our samples multiple times (“Recurrent”) was 90.1, while the percentage of calls that did not overlap which were “Recurrent” was lower at 83. This could indicate that there are more false positives in the copy-state 1 calls, and that calls are more likely to be observed in NGS studies if they are found in multiple samples.

Strain-matched comparison to the Sanger NGS data is somewhat limited, as the data include many call types that cannot be assessed with microarray analysis (as several classes of structural variation do not result in a large-scale dosage change). The types we did see overlap were tandem duplication (type H8 from [17]), duplication (type H10 [17]), nested deletion (type H11 from [17]) and deletions (type Del from [17]). Additionally, our results are not reported in relation to C57BL/6 J (as both NGS and aCGH are), but as relative to a diploid reference generated from all strains. This could lead to differences in strain-attribution of CNVs, and confound strain-matched comparison.

We also compared our calls to previous studies (Additional file 1: Table S5), without matching strains. The higher overlap percentage criterion ensures our CNV calls are not considered the same as small insertion and small deletion events (for example 1–50 bp), which are reported by NGS studies as SVs. A total of 5316 of our called regions have been seen previously in other studies, 8452 when including single base-pair overlap (Additional file 1: Table S5). Comparisons across array-based technologies are known to have low rates of concordance [9]. It is known that both the false positive and false negative rates are high for both aCGH and NGS [32]. The false positive rate for our CNV detection method is estimated to be between 15 %–25 %. Additionally, NGS studies to date have surveyed only 18 distinct mouse strains, which does not represent the diversity captured here. Increasing evidence for CNVs contributing to somatic mosaicism in human and mouse [7, 33, 34] is also consistent with discordance, as the tissue(s) chosen differed.

Another type of SV is that of mouse gene retrocopy insertion polymorphisms (GRIPs), which are retrotranspositions of processed mRNA transcripts, causing a copy of the source gene to be inserted (typically lacking introns and promoters) in one or more individuals, but absent from the reference genome [35]. The MDGA has only 14 probes that directly query GRIPs of the known 714 GRIP positions. Nevertheless, 152 of our CNV calls overlapped GRIP positions by at least 1 bp, representing 55 reported GRIPs from the autosomal reference genome. When considering instead, the 562 unique source genes of these GRIPs on the autosomes (545 of which contain probes), 467 of our calls overlapped with 80 of the GRIP source genes. Only 4 of these source genes correspond to a gene in the set of 55 found using the insertion site. Further study at higher sensitivity would be required to validate if these 614 CNV calls found by insertion site or source gene CNV are indeed GRIPs, or CNVs that span the same insertion sites or source genes.

### Recurrent CNV regions detected

Recurrence can arise from common inheritance or hotspots of mutation [36, 37]. We identified recurrent CNV events between samples as events with at least 40 % reciprocal overlap using HDCNV [38]. There are more recurrent events than unique events (Fig. 1, Table 2), but unique events affect more of the genome (123 Mb, or 4.98 %) than recurrent events (51.5 Mb, or 2.08 %). Recurrent events contain proportionately more genes than unique events (Fig. 1). There are 890 different regions containing recurrent CNV events, with a median of 3 events per region and 165 of these regions contain 10 or more recurrent CNV events.

**Table 2:** Number of unique or recurrent CNV calls on the autosomes by mouse classification

Mouse classification	CNV occurrence ^a^				Unique/recurrent
	Unique		Recurrent		
All	2418	(7.24)	7216	(21.60)	0.34
Classical	576	(5.05)	2248	(19.72)	0.26
Wild derived	870	(16.73)	1741	(33.48)	0.46
Wild caught	453	(23.84)	516	(27.16)	0.88
C57BL/6 J	12	(1.5)	78	(9.75)	0.15
C57BL/6NJ	0	(0)	46	(7.6)	0.00

Uniqueness and recurrence (found in two or more mice) are both consistently based on the entire analysis and are not reevaluated within mouse classification types (classical, wild derived, etc.), i.e. a call being unique in the wild-caught group was not found to overlap with any other call in the entire analysis and is not only unique within the samples classified as wild caught. In brackets, the call count is normalized by sample size

^a^A call is considered recurrent if it has 40 % reciprocal overlap with any other call

**Fig. 2:**
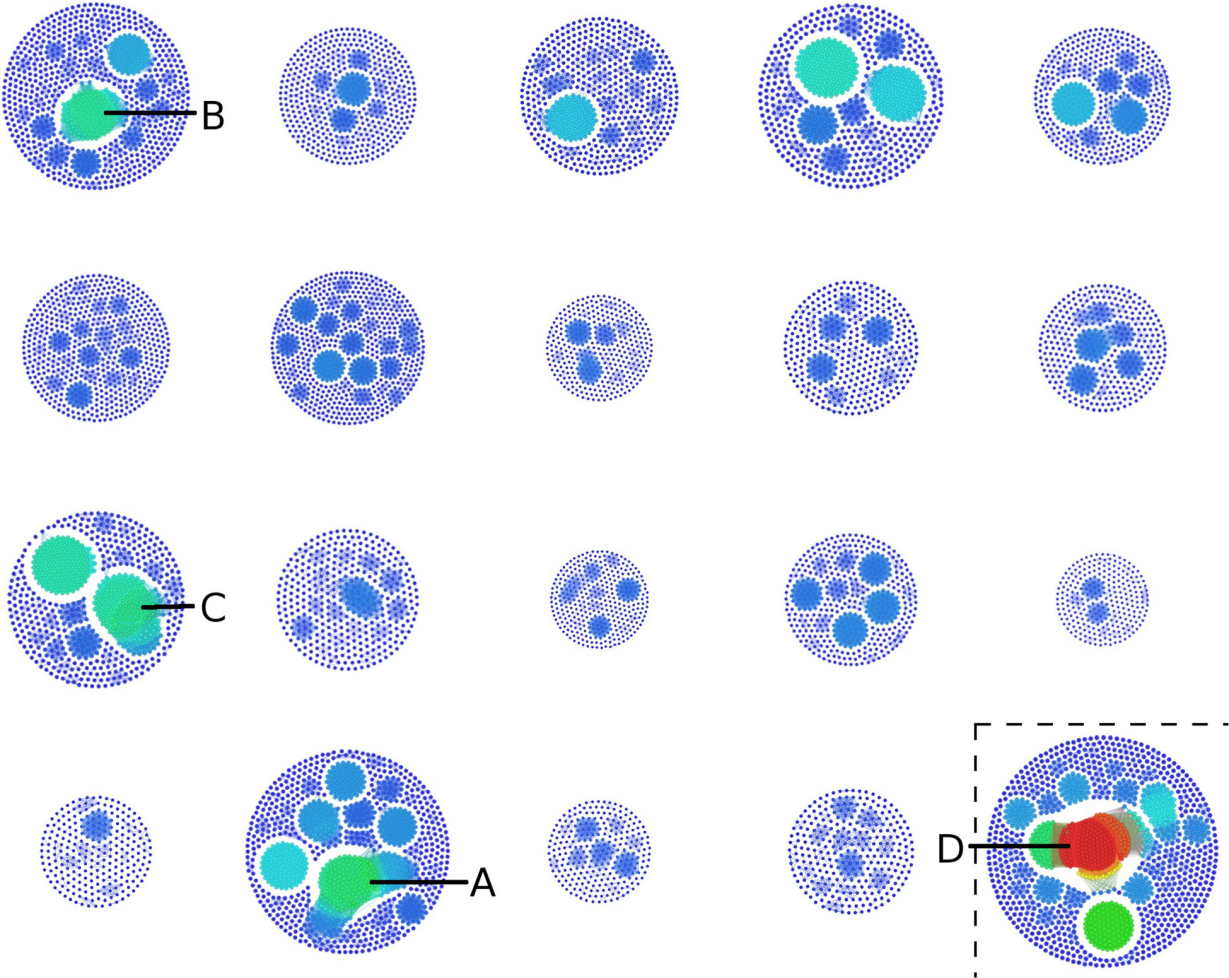
Number and recurrence of CNV calls. Each CNV call is represented as a single dot within larger circular clusters, with each cluster representing the autosomes 1–5, then 6–10 and so on. Calls with at least 40 % reciprocal overlap are joined by a line and considered recurrent. Each dot is then coloured on a heatmap scale, based on how many overlaps that call has with other calls on the same chromosome. The heat map colours range from 0 overlaps (dark blue) to 175 overlaps (red, chosen as it is half the number of total samples present). The total size of each chromosomal cluster is proportionate to the number of events found on that chromosome. Larger collections of connected dots represent CNV calls that are found in many samples, while unconnected dots represent unique events not shared among any samples. Labels A through D indicate complex clusters

**Fig. 3:**
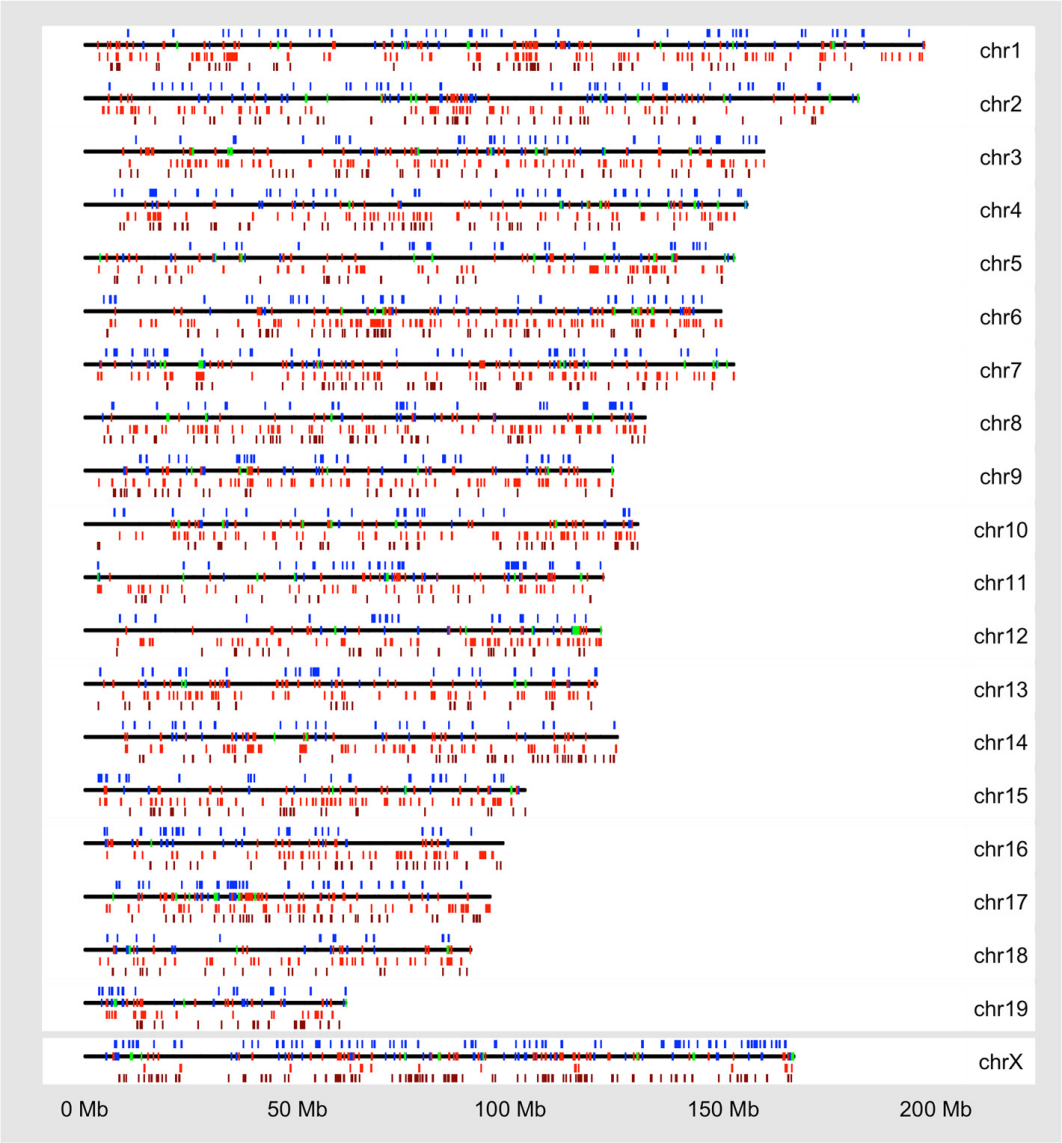
Copy number variants identified. For each chromosome both unique calls and recurrent regions are plotted. The unique calls are plotted for each chromosome as follows (listed from top to bottom): copy number amplification calls for three or more copies are plotted in dark blue above the region of the chromosome where they are found, the chromosome line in black, followed by one copy deletions in light red and full deletions in dark red below the chromosome line. The regions of recurrent CNV calls are plotted directly on the black chromosome line. Here, if the overlapping calls were all deletions, they are plotted in red; If they were all amplifications they are plotted in blue; If they are a mix of amplifications and deletions they are plotted in green

In Fig. 2, the relative number of calls per chromosome, as well as the degree of recurrence on each chromosome is shown, while Fig. 3 shows the locations and composition of recurrent events. The number of events per chromosome is not correlated to chromosome length, and the degree of recurrence varies between chromosomes. The copy number state of events within recurrent regions can be a mixture of amplifications and deletions (see green regions in Fig. 3).

The proportion of unique events seen in wild-caught mice is higher than any other classification subgroup (Fig. 1; Table 2). The high percentage of CNV-affected genome in our analysis (6.87 %) relative to other studies is likely due to the inclusion of wild-derived and wild-caught samples because these subgroups have more calls that are unique (Table 2). Classical laboratory strains and crosses with these strains, including the BXD strains (a recombinant inbred panel of C57BL/6 J and DBA/2 J) [39], the Collaborative Cross (CC:UNC) strains (a recombinant inbred panel of eight founders, five classical laboratory and three wild-derived strains) [40] and the F1-Hybrids (crosses of classical and/or wild-derived strains), all show a high proportion of recurrent CNVs (Fig. 1).

Some genomic regions contain recurrent events found in many of the samples. The region on chromosome 17 (label A; Fig. 2, Table 3) was characterized by NGS [14] as complex: a tandem duplication with a nested deletion. Here, the region contains a mixture of amplifications and deletions, with the deletions typically found in wild-caught samples, whereas the amplifications were typically found in classical laboratory strains (Additional file 1: Figure S2). This could indicate the same SV may be actually present in all samples, but array-based methods are not yet able to characterize this type of variation, or that this region is prone to multiple types of variation, which differ based on genetic background [19, 28]. Within the proximal third of chromosome 17 (the T region [41]), we identified 614 CNVs and most of these are recurrent (576 of 614). Amplifications in this region are more frequent than deletions (410 compared to 204) and there are 31 state-zero CNVs. An event was also found in a previous report at the regions seen on chromosome 1 (label B; Fig. 2, Table 3) which was a copy number amplification with a nested deletion or inversion [14]. Other events that appear similarly complex on chromosome 11 (label C; Fig. 2, Table 3), were characterized previously as deletions [14]. The complexity here is likely due to the difficulty in resolving correct breakpoints with the method used, but may indicate a site with multiple events arising from different mutations.

**Table 3:** Most common CNVs and complex^a^ CNV regions

Genomic location	Region type	Number of mice affected^b^	Gene symbol (gene type)^c^
17:6635443-6646618	CNV	66	*Tmem181c-ps* (ps)
14:44540155-44579921	CNV	43	*Ang5* (pc), *Ear-ps7* (ps), *Ear-ps10* (ps), *Ang6* (pc)
17:35383895-35392718	CNV	41	*Ddx39b* (pc), *SNORD83* (snoRNA), *CR974466.1* (miRNA)
11:116603748-116629092	CNV	40	*St6galnac1* (pc), *Gm11735* (ps)
4:122366514-122382286	CNV	38	*9530002B09Rik* (pc)
7:111681502-111683670	CNV	35	*Trim30e-ps1* (ps)
4:111790559-111972640	CNV	35	*Skint4* (pc), *Gm12820* (ps), *Gm12815* (ps), *Skint3* (pc)
17:30593663-31058945^d^	CNV	34	*Btbd9* (pc), *Gm9874* (pc), *Glo1* (pc), *Dnahc8* (pc)
5:114856193-114895051	CNV	34	*Ube3b* (pc), *Mmab* (pc), *Mvk* (pc)
14:20443929-20587951	CNV	34	*Gm17030* (ps), *Nid2* (pc)
17:30508869-31058945^d^	Complex	216	*Gm9874* (pc), *U6* (snRNA), *Glo1* (pc), *Dnahc8* (pc), *Glp1r* (pc), *Btbd9* (pc)
1:90097201-90210184	Complex	130	*Dnajb3* (pc), *Ugt1a5* (pc), *Ugt1a2* (pc), *Ugt1a1* (pc), *Heatr7b1* (pc), *Hjurp* (pc), *Ugt1a10* (pc), *Ugt1a9* (pc), *Ugt1a8* (pc), *Ugt1a7c* (pc), *Ugt1a6a* (pc), *Ugt1a6b* (pc), *Trpm8* (pc)
11: 70955732-71137889	Complex	152	*SNORA17* (snoRNA), *SNORA17* (snoRNA), *Nlrp1a* (pc), *Nlrp1b* (pc), *Nlrp1c*-*ps* (ps)

^a^ A complex region is defined as having boundary concordance below 0.75 as described in Cahan [7]. CNV events have exact boundary concordance

^b^ For CNV events, each mouse with the CNV is counted. In complex regions, a mouse is counted if they have any CNVs in this region and are not counted twice if more than one CNV in this region is present

^c^ Gene names are as in Mouse Genome Informatics Symbol. Gene types are one of: Protein coding (pc), RNA type as listed, or pseudogene (ps)

^d^ This CNV event is contained within this complex region

Within the wild-caught mice, CNVs that are present in more than five individuals of the same subspecies (of either *domesticus* or *musculus*) and not found in the other subspecies were identified (Table 4). Of the six CNVs identified as putative private variants in the wild *M. m. domesticus* population, only three are seen in any classical laboratory strain (overlap by 1 bp), while all six are seen in the wild-derived strains. Similarly, of the eight putative *musculus*-specific variants, only five are seen in the classical laboratory strains, while all eight are seen in the wild-derived strains. For the wild-derived strains 7.3 % of the calls overlap with these putative private mutations, whereas 2.5 % of the classical laboratory strain calls are in these regions. From this, it appears that for CNVs, the wild-derived strains capture more of the diversity seen in wild populations with respect to CNVs than classical laboratory strains.

**Table 4:** CNVs only in either wild-caught *M. m. domesticus* or *M. m. musculus* subspecies

Genomic Location			Type	Number of samples	Gene overlap
1:	28084515	−28126393	Del	5	-
2:	71652530	−71687549	Amp	5	*Itga6*
4:	121036609	−121090109	Del	5	*GM12866*
14:	10031933	−10032515	Del	5	-
18:	7348782	−7356220	Amp	8	*Mpp7*
19:	25900801	−25901740	Del	5	-
1:	90108589	−90166813	Amp	7	*Ugt1a, Heatr7b1, Hjurp*
4:	122366514	−122382286	Del	7	*9530002B09Rik*
4:	137702213	−137772702	Amp	5	*Eif4g3*
6:	142975631	−143048578	Amp	5	*C2cd5*
7:	18883984	−18892209	Del	5	*-*
7:	92886425	−92976094	Del	7	*Vmn2r72-ps*
8:	82175129	−82201642	Amp	9	*Otud4*
17:	31316283	−31478341	Amp	5	*Tmprss3, Ubash3a, Rsph1, Slc37a1*

### Enrichment of genomic features in CNVs

CNV regions were assessed for overlap with several genomic features and significant enrichments and depletions were identified by reshuffling the CNV calls 1000 times to build bootstrapped 95 % confidence intervals. Amplification regions are enriched for CpG islands which were found in 34.8 % of amplification calls. In contrast, deletions are depleted for CpG islands, overlapping only 6.9 % of the deletion calls (Additional file 1: Figure S5). CpG islands are also enriched in 500 bp and 1 kb regions spanning the ends of both deletion and amplification calls (Additional file 1: Figure S6). CpG islands were shown to be overrepresented in CNV breakpoints in humans [29]. While GC content would increase the binding affinity for probes in GC rich regions, GC-model correction was performed prior to CNV detection, which should have mitigated or eliminated this source of bias [42].

CNV regions 10 kb and larger are enriched for segmental duplications, as in Cahan *et al.* [7], with 40.4 % of these calls overlapping annotated segmental duplication regions, approximately double that expected by chance (Additional file 1: Figure S5). Segmental duplication overlap is also enriched in the 500 bp, 1 kb and 2 kb regions centered around the endpoints of the 10 kb and larger CNV calls (Additional file 1: Figure S6), as was seen for the breakpoints of SVs not associated with transposable elements found by Quinlan *et al.*[12]. These findings support the emerging evidence that segmental duplications may cause local genetic instability, resulting in structural alterations like CNVs [12].

A large portion of structural variation in the mouse genome is driven by transposable elements including short interspersed nuclear elements (SINEs), long interspersed nuclear elements (LINEs) and long terminal repeats (LTRs) [11, 12, 16], but these regions are not well captured by array technologies because of the inherent difficulty in designing suitable probes for repetitive elements. In the probe sets used here, which were confirmed to have all probe sequences uniquely aligning to the reference genome, only 4.9 % of the autosomal probes directly query annotated LINE, LTR or SINE events.

The LINE and LTRs are present in a high proportion of CNV calls (80.2 % and 80.1 % respectively) but are depleted when compared to the randomly resampled genomic regions. A lower proportion of amplification calls was found to overlap these elements in the classical laboratory strains (74.6 % and 75.6 %) compared to all amplification calls (81.5 % and 82.8 %), as well as compared to both the wild-derived and wild-caught classification subgroups (Additional file 1: Figure S8). A higher number of variants associated with transposable elements were found in several wild-derived strains compared to the C57BL/6 J reference by Nellåker *et al.* [16], which would present as an amplification (or deletion, as the reference here is not C57BL/6 J) overlapping an annotated repeat element in our results. The higher proportion of LINE- and LTR-overlapping amplifications in wild-derived and wild-caught strains is therefore consistent. A lower proportion of deletions was found to overlap with LINE and LTR elements in classical laboratory strains as well, though the difference is not as pronounced.

When considering only calls of 10 kb or greater, LINEs and LTRs are enriched in deletions, while depleted in amplifications of this size, and are depleted in all calls below 10 kb in length (Additional file 1: Figure S5). Conversely, CNVRs of 10 kb and larger were enriched for LINEs and all CNVRs showed enrichment for LTR by Cahan *et al.* [7]. All repeat elements showed enrichment in Yalcin *et al.* [14]. SINE elements, as expected, are enriched in all CNV calls, with a higher proportion of amplifications overlapping these regions (86.2 % of deletions, 95.0 % of amplifications) (Additional file 1: Figure S5).

In several studies, the proportion of LINE and LTR elements was greater than the SINE elements [11, 12, 16], which is not the case here. It should be noted that here we report overlap with annotated elements. Another confounding factor is that while 4.9 % of the probes used target LINE, LTR or SINE annotated genomic reference DNA, 30.3 % target intronic regions and 47.8 % target exonic regions. This may explain why we see a depletion in LINE and LTR regions (as they have been shown to be depleted in intronic and exonic regions [16]) and high proportions and enrichment of SINE elements (which tend to reside in regions with high GC content and have been shown to be enriched in intronic and flanking regions of genes [16]).

The start and end positions of CNV calls reported here are the genomic positions of the first and last probe contributing to the CNV call. With the median inter-probe-set distance of 319 bp, it is not possible to identify exactly the breakpoint of the event, let alone the sequence surrounding the event, which confounds mechanistic analysis or inference. While we do see similar results to that of other reported mechanism-related findings, this is only from investigating the trend across all samples, rather than classifying a putative mechanism for each specific call in a sample. Mechanistic classification is better characterized using sequencing methods [12, 14, 16], or targeted arrays of much higher resolution [29].

### Gene content of CNVs

The majority (65.7 %) of CNVs entirely encompass at least one gene, are entirely encompassed by a gene or partially overlap with at least one gene (Fig. 1). The proportion of CNVs containing protein-coding genes in the classical laboratory mice (76.7 %) is higher than in the wild-caught mice (54.2 %). The three main Ensembl classification types, excluding regulatory elements, for regions that overlap CNVs are protein-coding genes (76 %), pseudogenes (11 %) and multiple classes of RNAs (10 %).

Protein coding genes were found in a higher proportion of amplifications (88.8 % of amplification calls overlapped a protein coding gene region) than deletions (55.6 %). Pseudogenes were also found to overlap a higher proportion of amplifications (18.0 %) than deletions (13.9 %), as were RNAs (18.2 % vs 7.1 %) and antisense gene regions (5.1 % vs 2.6 %). As expected, it is less likely to find deletions in these regions as they are likely to be deleterious [43].

The most common CNV (when considering events with the same start and end position in each sample) is in 66 mice on chromosome 17 and contains the *Tmem181c-ps* pseudogene (Table 3). Almost all (93 %) classical laboratory mice with this CNV have an amplification, while all wild-caught mice have a single-copy deletion. The second most common CNV (Table 3) contains two pseudogenes, *Ear-ps7* and *Ear-ps10*, as well as two protein-coding genes, *Ang5* and *Ang6*. This CNV was observed only as a copy number state of either 0 or 4 and both states existed in classical laboratory and wild-caught mice subgroups. This CNV occurred most frequently in the BXD subgroup.

CNV differences were observed in samples from the same mouse strain. Six of eight C57BL/6 J mice have an extra copy of the insulin-degrading enzyme (*Ide*) gene and half of the C57BL/6 J mice have an extra copy of the fibroblast growth factor binding protein 3 (*Fgfbp3*) gene. None of the C57BL/6NJ mice have the *Ide* or *Fgbp3* amplification. Watkins-Chow and Pavan [44] identified an increased copy number in *Ide* and *Fgfbp3* in a large proportion of the C57BL/6 J mice that results in increased gene expression.

All eight C57BL/6 J mice in our study also have CNV amplifications overlapping most of *Skint4*, *Nlrp1b* and *Slc25a37* although none of these genes were encompassed completely by a CNV like *Ide* and *Fgfbp3*. Single-copy deletions overlapping *Gm9765* and *Btbd9* are also common (found in > 50 % of samples). The *Skint4* two-copy amplification is also in all six C57BL/6NJ mice. Our data continue to support intrastrain CNV differences as important contributors to divergence from isogeneity [42].

CNV amplifications that overlap with *Skint4*, *Nlrp1b*, *Slc25a37*, *Ide* and *Fgbp3* in our study were called as CNV deletions in non-C56BL/6 J laboratory strains in previous studies [3, 4, 6–9, 13, 45]. Similarly, the CNV deletions in *Btbd9* were called as CNV amplifications in previous studies [3, 4, 7, 9, 14, 45]. *Gm9765*, which appears as a deletion in our C57BL/6 J mice, appeared as a amplification in inbred mice in six other studies [3, 7, 9, 13, 14, 45] while one study found a mix of deletions and amplifications in this region [8]. This may indicate that the CNVs overlapping with these six regions (excluding *Gm9765*) are widespread in the C57BL/6 J mouse and using this mouse strain may result in incorrect CNV states reported in other strains.

When only considering genes completely encompassed by CNVs and CNVs completely encompassed by genes (complete overlap), the top gene enrichment terms differed between wild-caught and classical laboratory mice. Across classical laboratory mice, only the gene ontology (GO) terms for amplifications are significant, while in wild-caught mice, GO terms for both deletions and amplifications are significant (see Additional file 5). The most significant GO term across classical laboratory mice is ‘antigen processing and presentation of peptide antigen’ (*P*_*adj*_ = 3.26 × 10^−10^). Most of the top GO terms for classical laboratory mice are related to immunity or structural organization of the genome. Laboratory mouse strains are frequently bred to display specific immunity or disease phenotypes and this may in part explain the GO term enrichment across the classical laboratory mouse strains for immunity-related terms [46]. Across wild-caught mice, GO terms related to olfaction are significant for deletions while GO terms related to pheromone response are significant for amplifications. Olfaction- and pheromone-related genes, which can assist mice with social interactions and gaining information about their environment [47], are not highly enriched in GO term analysis for classical laboratory mouse strains, consistent with their laboratory breeding history. Similar to CN variation, SNP variation in pheromone receptors is lower in classical laboratory mice when compared to wild-derived mice [48].

Ingenuity Pathway Analysis (IPA) gene groupings into top diseases and functions networks show differences between wild-caught and classical laboratory mice for CNVs completely within or completely containing a gene, although the distinction isn’t as clear as with DAVID (see Additional file 6). A total of 45 networks with an IPA score not less than 10 were identified. More networks are affected by amplifications (28) than by deletions (17) and, in particular, by amplifications across the classical laboratory strains (22).

An overrepresentation of lipid metabolism genes has been shown in CNV regions in wild-caught mice [8]. Here, ‘lipid metabolism’ is among the top biological functions for a network associated with amplifications across wild-caught mice and is not found for CNVs in classical laboratory mice. Conversely, classical laboratory mice have a network associated with ‘carbohydrate metabolism’ in amplifications, as well as ‘amino acid metabolism’ in one-copy deletions. This difference may indicate CN variation as an adaptive change to diet between wild-caught mice and classical laboratory strains. In humans and dogs, the copy number of the amylase (*AMY1, AMY2B*) gene was found to vary and in dogs is also found to be amplified over wolves, conferring adaptation to a starch-rich diet [49, 50]. Across all of our samples, there is only one amplification in the mouse ortholog to these genes (*Amy1, Amy2*), found in the YBR/EiJ classical laboratory strain, so there is no evidence for an adaptive change to diet involving CNVs in the mouse amylase genes within our sample mouse population.

Development terms were found in 23 of the 45 networks associated with CNV regions and included cellular development, tissue development and the development of a variety of systems (for example neurological, hematological, gastrointestinal, etc.). For all genes present in the mouse (Ensembl:67), 34 out of 50 of their associated networks when analyzed as a whole with IPA include development terms, so this result is not unexpected.

Across mouse strains, networks involved in ‘endocrine system development’ are associated with amplifications in wild-caught mice and with state-zero deletions in classical laboratory mice. Networks involved in ‘cardiac system development’ are only associated with amplifications in classical laboratory mice and not associated with CNVs in wild-caught mice. Networks involved in ‘inflammatory response’ are associated with CNVs (both in deletions and amplifications) in the classical laboratory mice, but not in the wild-caught mice. Networks involved in ‘cell mediated immune response’ were found to be associated with amplifications in both classical laboratory and wild-caught mice.

CNV calls may differ by strain due to strain-specific SNPs preventing the hybridization of probes and the target DNA. Subsequently, there will be a bias in the gene enrichment depending on how closely related a mouse is to the probe design reference.

### Genes unlikely to harbour copy-number deletions

CNVs were not found in 26 gene regions that are conserved across mammalian species and which have been used to construct phylogenetic trees (Additional file 1: Table S6) [51]. Autosomal genes *Adam17, Cdk8, Col7a1, Dll1, Dnmt3b, Dyrk1a, Eed, Eln, Ezh2, Igf1, Lama5, Med1, Med24, Med21, Med30, Pex7, Pknox1, Pdpk1, Slc2a1, Suz12*, *Vps35* and *Tfrc* are known to cause deleterious phenotypes when gene expression levels are reduced and may be lethal at a zero-copy state or one-copy state, depending on the gene [52–74]. Therefore, deletions in these gene regions, particularly state zero deletions, are not expected. Three mice appear to have partially lost one copy of *Col7a1*. Unlike a zero-copy deletion, a single-copy deletion of *Col7a1* is not lethal. Mice in this latter case are expected have a normal phenotype if gene expression levels are high enough [56]. As expected, no deletions were detected in any of the other autosomal genes listed above.

A number of genes on the X chromosome cause deleterious phenotypes when deleted, including *Aifm1, Alas2, Amer1* (synonyms *Wtx* and *Fam123b*)*, Bcor, Cask, Cul4b, Ebp, Flna, G6pdx, Gyk, Ikbkg, Mecp2, Med12, Mtm1, Nsdhl, Ofd1, Piga* and *Porcn* [75–94]. Two male mice from our analysis are partially missing the *Cask* gene (approximately 33 % and 6.5 % missing). Although a knockout of *Cask* is lethal, mice are still viable even if *Cask* expression has been reduced by ~70 % [84]. SINE and LINE deletions, up to 4761 bp in size, have been found in *Cask* [11] and a large CNV deletion covering the entire *Cask* gene was identified in an aCGH study [95]*.* As long as some degree of the functioning *Cask* gene is maintained in the mouse it is possible for *Cask* to acquire mutations or be lost in a cell population.

There are several possible explanations for observing CNV deletions that overlap genes that when deleted contribute to deleterious phenotypes. The deletions could be false positive calls or could be due to off-target mutations in the samples which prevent probes from binding in these areas. Detecting deletions deleterious to a mouse may be biologically viable, and examples of somatic variants, in the tissue sampled given that gene's developmental stage-specific or tissue-specific expression. Previous reports have also found several genes overlapping non-retrotransposon-related deletions over 500 bp from previous reports for the autosomes [8, 11–13]: *Adam17, Cdk8, Dnmt3b, Dyrk1a, Eed, Ezh2, Lama5, Med21, Pdpk1* and *Pex7*, as well as on the X chromosome [3, 11, 13]: *Aifm1, Bcor, Cask, Cul4b, Ebp, Flna, G6pdx, Gyk, Ikbkg, Mecp2, Nsdhl* and *Porcn*. Single-copy losses or minimal expression of these genes can be tolerated in mice. Previous reports do not list an integer copy number state, so it is possible that the reported deletions in these gene regions are one-copy-state deletions.

### Comparison of SNP and CNV distance

**Fig. 4:**
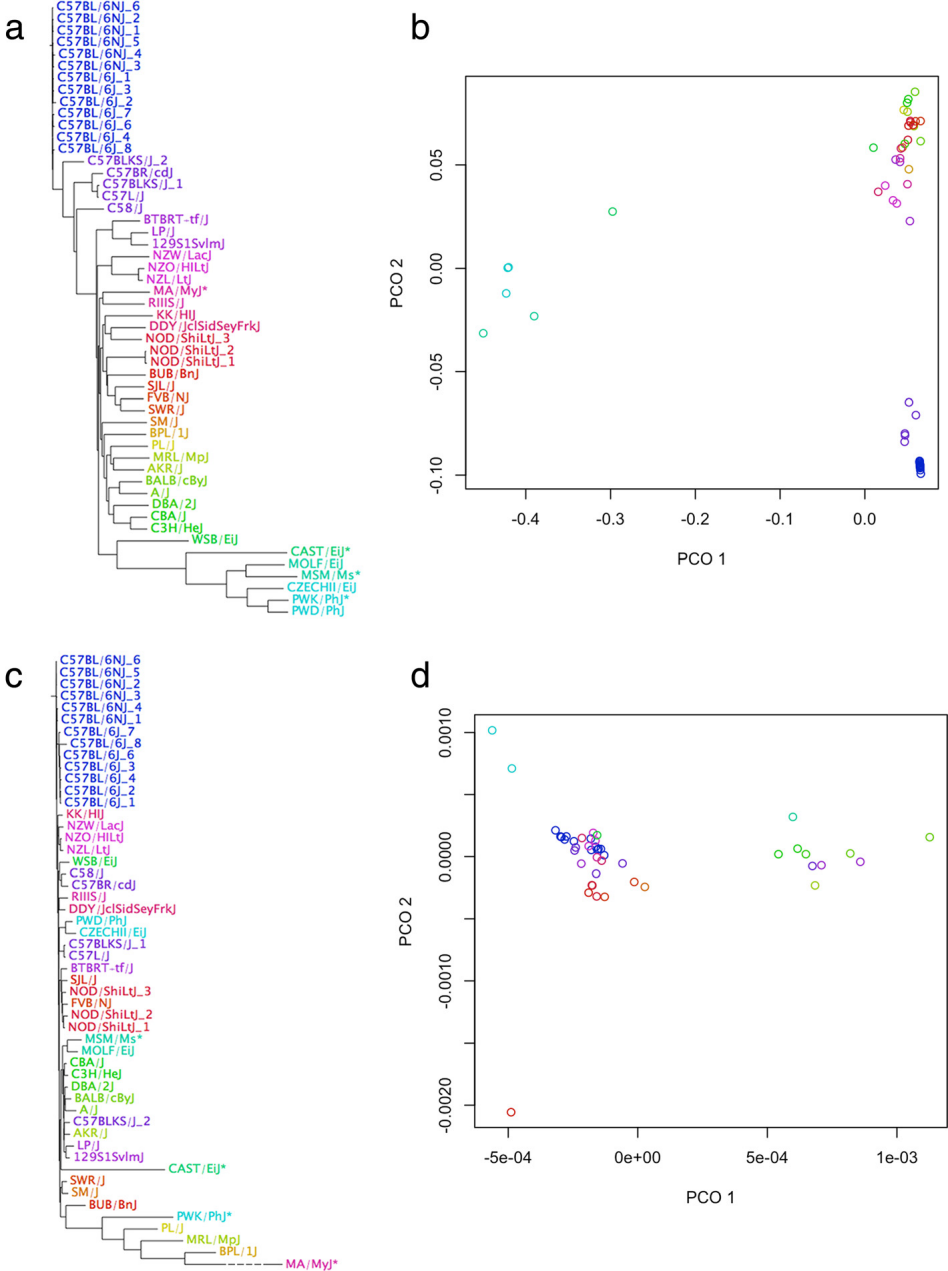
SNP and CNV distance for MGI priority and 17 genomes project strains. **a**. Neighbor joining tree constructed using SNP distance. **b**. Multidimensional scaling (MDS) for SNP distance matrix, showing first two principle coordinates. **c**. Neighbour joining tree constructed using CNV distance. Trees are not proportional to each other. The dashed line for MA/MyJ indicates a manually shortened branch. **d**. MDS for CNV distance matrix. All diagrams are coloured based on similarity in SNP distance (panel **a**)

After calculating the SNP and CNV distance by probe set for the MGI priority strains and the strains from the 17 genomes project, we generated trees using neighbor joining [96] (Fig. 4a/c) and performed multidimensional scaling on the distance matrices to find the first two principal coordinates (Fig. 4b/d).

The main source of variation in the SNP distance (PCO1) can be explained by the presence of a *Mus musculus musculus* subspecific background (for the MOLF/EiJ, MsM/Ms, CZECHII/EiJ, PWK/Phj, PWD/PhJ strains) and *Mus musculus castaneus* background (for the CAST/EiJ strain) [23]. The second source of variation (PCO2) somewhat follows the percentage of *Mus musculus domesticus* background. Yang *et al.* [23] showed that the A/J strain’s genome is approximately 96.6 % *domesticus* in background, whereas the B6 mice are approximately 92.8 %. These samples are the high and low extremes of the second principle coordinate (PCO2) respectively (Fig. 4).

The CNV distance measure does not replicate SNP distance directly. The distance matrices for the SNP and CNV calls are significantly positively correlated for the strains shown in Fig. 4d (*P* < 0.0001, Mantel-test statistic 0.61). The positive correlation indicates that CNVs may follow a similar pattern of relatedness to SNPs, though do not directly recapitulate the same distances between strains. Biologically, *de novo* CNVs would contribute to confounding the relatedness of individuals, though technical error is also a likely contributor to this discrepancy. The SNP calls are less likely to be affected by technical error as they rely on relative intensity within a probe set, rather than a consecutive group of increased or decreased intensity values relative to a reference, which makes CNV analysis more susceptible to hybridization variation between chips and erroneous calls. This may explain why the SNP trees somewhat follow expected structure, while the CNV tree has higher as-yet-unexplained variation.

Several samples were excluded from Fig. 4d, as these samples were the overwhelming source of variation in both the 1st and 2nd principle coordinates in the analysis when included (Additional file 1: Figure S8). The samples removed either fail quality control for CNV calling (CAST/EiJ, PWK/PhyJ, MA/MyJ) or were the top three highest number of calls in the passing samples (BRL/1J; 94 calls, MRL/MpJ; 89, PL/J; 76). The exclusion of samples for having a higher-than-expected number of copy number calls was done in a similar study using human samples as a quality control measure [97], where individuals above the 95th percentile for CNV number were excluded, a cutoff that the samples excluded here also meet.

Off-target mutation can result in low call rates during genotyping, as seen in the work with VINOs [24] and may also affect CNV calling. While the Affymetrix^®^ Power Tools genotyping software does not output VINO calls, it will call most VINOs as either heterozygous or as “no calls” [24]. The difference in the SNP “no call” rate within CNV calls by strain between passing and failing samples is not significant. It is significantly different, however, between wild-derived or wild-caught samples (mean 3.04 %) and all other samples (mean 1.09 % : *P* < 2.2 × 10^−16^; Wilcox rank sum test). The deletion rate is higher in these subgroups across the entire sample set of 351 mice, but it should be noted that the same trend is seen in amplification calls between these same subgroups (no-call rate mean 0.83 % and 0.41 % respectively: *P* = 1.235 × 10^−11^; Wilcox rank sum test). The same trend is also seen in the heterozygous call rate (means 1.25 % and 0.87 % respectively: *P* = 1.037 × 10^−7^; Wilcox rank sum test), which could indicate that these subgroups have a high rate of false positives, as we do not expect heterozygous calls in deleted regions.

### Droplet digital PCR confirmation of select genic CNVRs

For a total of 252 ddPCR confirmation assays, 242 of those reactions (96 %) were in agreement with a predicated CN state made based on a different mouse of that strain (Additional file 1: Tables S7 and S8). There were no discordant CN calls among the ddPCR technical replicates. Two of the selected CNVRs are known to contain genes *Ide* and *Fgfbp3* that vary in copy number within the C57BL/6 J mouse strain [44]. The within strain differences in CN state for the gene *Fgfbp3* were also observed among the ddPCR assays for the 5 C57Bl/6 J mice tested. The inter-strain differences in CN state for select genic CNVRs affecting *Hdhd3* and *Skint3* and *Glo1* genes were also confirmed by ddPCR. Three of nine ddPCR gene assay results (*B4galt3, Ide* and *Fgfbp3*) matched the predicted CN state for all three mouse strains. *Skint3* and *Trim30e-ps1* CN states were zero for all CBA/CaJ and DBA/2 J mice when a CN state of two was predicted. For *Skint3*, a CN state of zero has been reported for DBA/2 J [98]. The CN gains predicted for *Hdhd3* in CBA/CaJ and DBA/2 J mice were detected by ddPCR and called as a CN state of six in both strains. *Skint3* ddPCR CN states were found to be increased by one state for all three mouse strains when compared to the predicted states. Contrary to predictions, *Itln1* ddPCR determined CN states did not differ from two in the five C57BL/6 J mice tested. Notably, any differences from the predicted CN states are not necessarily indicative of MDGA performance given that different mice were used for the microarray and ddPCR based determinations.

### X chromosome analysis

CNV detection on the X chromosome was performed using the samples’ known sexes to establish an expected baseline copy number state. Male mice have a much higher incidence of calls per mouse on the X chromosome when compared to female mice and have fewer deletions than amplifications, whereas female mice have about an equal number of deletions and amplifications (Additional file 1: Table S9). Similar to the autosomes, there are more calls per mouse in the wild-derived and wild-caught mice than in the classical laboratory strains. The ratio of deletions to amplifications increases in wild-derived and wild-caught mice. The X chromosome has a large number of recurrent calls (Fig. 2). This region overlaps the *Mid1* gene found at the distal end of the X chromosome in the pseudoautosomal region, known to have a high frequency of unequal crossovers [99]. In general, the unique to recurrent ratio is lower for all classes than is seen for the autosomes, with the classical laboratory strains having the fewest unique calls per mouse.

When compared to previous reports, only 423 calls overlap reciprocally by at least 20 % (317 of those with aCGH studies, 356 NGS) and 984 by at least 1 bp (520 aCGH, 942 NGS). The majority of CNV deletions (~59 %) do not overlap with protein- or RNA-coding regions of the X chromosome or with pseudogenes, while the majority of CNV amplifications do overlap with these regions (~93 %). This observation was expected because gene deletions on the X chromosome are more likely to be deleterious than amplifications. The most highly enriched GO terms for CNVs on the X chromosome are generally related to chromosome organization and interaction with proteins. “Chromosomal part” is the most significantly enriched GO term for duplications (*P*_*adj*_ = 4.57 × 10^−2^) and “protein-DNA complex” is ranked highest for deletions (*P*_*adj*_ = 6.57 × 10^−3^). GO terms are not significantly enriched for genes overlapping with classical mouse CNVs or for wild-caught CNV amplifications. There are 19 significantly enriched GO terms (*P*_*adj*_ < 0.05) for genes in wild-caught mouse deletions, almost all of which were related to the organization of the genome.

### Caveats of MDGA for CNV detection

The false discovery rate as reported for PennCNV software is 9 % [100], however, this was calculated for calls from Illumina microarrays. Affymetrix^®^ microarrays, including the MDGA, use shorter probes (25 nt) and tend to be noisier than longer-probe chips. We estimate the false positive rate for deletions to be 22.5 % [101], similar to previous work with PennCNV on the Affymetrix^®^ Human 6.0 microarray which reported a 24 % false positive rate for deletions [102].

The number of calls per chromosome is positively correlated with the number of probes per chromosome (ρ = 0.78: *P* < 0.0001; Pearson correlation), which is an inherent ascertainment bias of probe-based technologies. Also, there are large regions of the genome for which no suitable probe sets can be designed (Additional file 1: Figure S9), or where probe sets are too sparse to result in CNV call passing our quality control measures. Also, as mentioned, there are more probe sets covering exonic and intronic regions than intergenic regions.

The 17 samples that did not meet the quality control cutoff were analyzed separately. Ten of these were wild-derived or wild-caught samples, including the MDGI, PWK Hybrid and PWK/PhJ mice, as well as the MSM/Ms, CAST/EiJ, DDO and four *M. m. musculus* individuals caught in Poland, Hungary and China. These mice would be expected to have a high amount of variation from the C57BL/6 J mouse for which the probe sets are targeted. Many of the CNV calls we see for these mice (30–175 calls with the majority of events being deletions) could errantly arise from their genomic DNA failing to hybridize strongly to a number of probe sets in a genomic region because they have diverged from the C57BL/6 J mice in those regions, i.e. have undergone multiple base substitutions or mutations, rather than copy number changes. A similar phenomenon may be underlying deletion calls in diverse samples that passed quality control cutoffs. Further biological confirmation would need to be performed to confirm if these are false positives or represent true variation in these strains.

Several studies have suggested the use of multiple algorithms used in consort to call CNVs in order to increase reliability and biological conformation [103, 104]. We have chosen to only use PennCNV, as it was the only package at the time of writing that could be adjusted to use the IGP probes present on MDGA to produce integer copy number. This is the first study to our knowledge to use the IGP probes. The comparison of calls made with both SNP and IGP probes, to those made only with SNP probes, would essentially negate the use of the IGPs in the PennCNV analysis. PennCNV has been shown to work well in other similar studies for Affymetrix^®^ microarrays using human samples [102, 105, 106].

To mitigate the effect of these caveats, biological validation, such as qPCR, would normally be performed using the same DNA samples but was not feasible in this study, both in acquiring the same samples used for the study (as it has been shown that the strains may not be isogenic, and are subject to somatic mosaicism), as well as the scale of the project. Herein, confirmation of select genic CNVRs in classical inbred strains is a first step toward biological validation and future work could be expanded for CNVs of biological interest. Also, as research progresses toward characterizing higher levels of diversity, both the probe choice and algorithmic methods must be adapted.

## Conclusions

The microarray is a valuable tool for large-scale analysis and when analyzed with rigour can provide insight into SNP and CN variation. Here, we used publically available microarray data and identified and characterized CN variation in a large sampling of *Mus musculus*, with 82.5 % of the calls reported for mice that had not been studied for CNVs. We provide several resources for researchers, including a probe list that has been filtered to avoid possible sources of noise in CNV analysis, a list of genes to use as a negative control in CNV studies as well as the CNV calls and strain information generated and analyzed here, all of which will inform future study.

We found differences in the genes affected by putative CNVs between wild-caught and classical laboratory mice, most notably in genes related to lipid, carbohydrate and amino-acid metabolism, as well as immunity, pheromone response and olfaction. This supports the hypothesis that CNVs play a role in increasing genetic diversity and have phenotypic impacts that when shaped by selective pressures confer adaptation.

With increasing research interest in somatic mosaicism, the mouse provides a direct way to analyze CNVs between tissues under a variety of controlled genetic backgrounds and environments. The mouse will continue to be a highly relevant model organism for understanding human development and disease, as an experimental system with a high level of control as well as tissue and cell type availability. Our findings provide the most comprehensive picture to date of CNVs in mice using microarray technology.

## Methods

### Samples

351 publically available Mouse Diversity Genotyping Array CEL files were downloaded from the Center for Genome Dynamics at The Jackson Laboratory [27]. These files contain raw array intensity data for mouse tail samples from 120 classical laboratory strains, 58 wild-derived strains, 10 consomic strains, 1 congenic strain, 44 BXD recombinant inbred strains, 40 CC-UNC G2:F1 strains, 55 F1 hybrids and 23 wild-caught mice.

### Assessment of probe suitability and annotation accuracy

SNP annotation files were filtered (Eitutis, unpublished). Original IGP annotation files were downloaded from the Center for Genome Dynamics website [27]. Invariant genomic probes (IGPs) that were classified as Exon 1 and Exon 2 were locally run through BLAST to ensure that the probe sequences were found only once in the mouse genome (UCSC:mm9) and to verify the annotated position. Probe sequences were verified as 25 bp in length, not duplicated by another probe sequence and having complimentary sense and antisense sequences.

In-house scripts removed probe sets likely to contribute to background noise and false positives, including those containing palindromic *Nsp*I or *Sty*I recognition sites within a given probe sequence and its 12 bp flanking region (as the genomic target sequence is digested by these restriction enzymes prior to hybridization to the array) as well as probe sets overlapping other probe sets based on genomic position, as these would compete for genomic DNA template (see Additional file 1: Figure S1). The SNP and IGP annotation files were further filtered to create a more stringent probe list, but this was after the CNV calling and analyses were completed (see Additional file 1: Figure S1, and Additional files 2 and 7). Due to the presence of large spans of the genome where no probes were present, inter-probe-set distance outliers beyond the third quartile were removed before assessing the median.

### CNV identification

Genotype calls were generated using the BRLMM-P algorithm implemented in Affymetrix^®^ Power Tools [107] using default parameters as specified by Genotyping Console, which includes quantile normalization. A canonical genotype clustering file was generated and used to calculate Log R Ratio (LRR) and B allele frequency (BAF) values using the PennAffy package [108]. PennCNV was used to generate a PFB (population frequency of the B allele) reference file from the data above [100]. A GC model file, containing the percent GC content of the 1 Mb region surrounding each marker (or the genome-wide average of 42 % if this could not be calculated) was generated using KentUtils [109] and an in-house script based on the reference genome (UCSC:mm9). CNVs were detected with PennCNV using default parameters and GC model correction [42]. CNVs on the X chromosome were detected in a separate run of PennCNV using the –chrX option. Calls were filtered to be 500 bp to 1 Mb, have at least three markers, have a marker density of 0.00013 markers/bp, have a log-R ratio below 0.35 and have a B allele frequency drift below 0.01.

### CNV analysis

The Sankey diagram was generated from annotated calls with the rCharts package in R using the d3.js plugin. Recurrent CNV calls were identified with HDCNV, using 40 % reciprocal overlap [38]. The graph files generated for each chromosome were formatted using Gephi [110] (Fruchterman-Reingold layout) and image manipulation software tools (sips Apple command line tool and ImageMagik [111]) were used to scale and combine the images. Individual chromosome images were scaled to be proportionate to each other using the number of calls as a proxy for their area.

### Concordance of CNV calls with previous reports

Data were downloaded from the Database of Genomic Variants [112] or from supplementary tables depending on availability. Overlap analysis at 20 % reciprocal overlap and at 1 bp overlap was performed using the intersect function of Bedtools (version 2.17.0) [113]. The copy number state of the call was not considered; the presence of a call in a previous study was considered evidence that variability occurs in this region.

### CNV mechanistic context

CNVs are considered to overlap a genomic feature if there is at least 1 bp of overlap. LINEs, SINEs, LTRs as annotated in the repeatMasker (rmsk) table, as well as CpG islands and segmental duplications were downloaded from the UCSC table browser [114]. To identify significant enrichments and depletions, CNV calls were reshuffled 1000 times within the chromosomes on which they were found to maintain chromosome and size distribution. A 95 % confidence interval for each feature was determined by running overlap analysis on the shuffled regions and to identify the 25th and 975th ordered number of overlaps. To assess putative CNV breakpoints, the 500, 1000 and 2000 bp regions surrounding the start and end position of each call were found and analyzed separately, removing any call where the flanking windows end up overlapping. Overlap and enrichment assessment were then performed as previously stated.

### Gene analysis

Gene annotations were downloaded from Ensembl BioMart [115, 116]. Genes found in CNVs were identified using in-house scripts. Ensembl genes were used for consistency with the original probe annotation files.

The Database for Annotation, Visualization and Discovery (DAVID) v6.7 [117, 118] Functional Annotation tool was used to identify GO term enrichment for genes overlapping CNVs. DAVID automatically excludes redundant genes from its analysis. The three default GO categories (GOTERM_BP_FAT, GOTERM_CC_FAT and GOTERM_MF_FAT) were used to identify the most relevant GO terms for each gene list. Occasionally, pseudogenes can be “resurrected” and produce translated products [119]. For this reason, pseudogenes classified as having a protein-coding biotype by Ensembl were included in the gene analysis.

Lists of genes were grouped into disease and biological function networks using QIAGEN’s Ingenuity^®^ Pathway Analysis’ Core Analysis [120]. Direct and indirect relationships with a maximum of 35 focus molecules per network were included. Human, mouse and rat genes were included. The confidence level was set to include experimentally observed relationships between focus molecules as well as predicted relationships that have a high confidence. Molecule relationships with endogenous chemicals were excluded.

### Genetic distance determinations and phylogenetic analyses

SNP distance was calculated pairwise for each sample by genotype call at each SNP locus. SNPs that cannot be assigned a genotype are returned as “no calls” and shared “no calls” for sample pairs were not considered a difference. To calculate CNV distance, the copy state of each sample’s call (0,1,2,3+) was assigned to each probe sets positioned within those calls, then pairwise differences between samples were counted for each probe set and divided by the total number of probe sets. The tree was generated with the APE package (version 3.0-11) for R (version 3.0.2) using the bionj function which uses the minimum evolution algorithm of Desper and Gascuel [121]. The tree images were created and coloured using FigTree (version 1.4.0). Multidimensional scaling on the distance matrices was also performed using “cmdscale” function in R and plotted. Mantel’s tests were performed using the “mantel” function from the vegan package (version 2.0–10) in R.

### False discovery rate

To estimate the false discovery rate in our CNV calls, the method of Baross *et al.* [101] was applied. The genotype calls for SNP markers are expected to be homozygous if they fall within a detected deletion CNV (both zero- and one-state-copy deletions). Calls in which more than 10 % of the genotype calls are heterozygous are considered as false positives.

### Select genic CNVR confirmation by droplet digital PCR (ddPCR)

Nine genic CNVRs found in C57BL/6 J mice were selected for CNV confirmation by ddPCR in five C57BL/6 J, five CBA/CaJ and four DBA/2 J inbred mice (see Additional file 1: Table S7). For each CNVR, one TaqMan^®^ Copy Number Assay (Thermo Fisher Scientific, Waltham, Massachusetts, USA) was selected for a gene overlapping that CNVR. Overall, nine gene assays were conducted for the 14 mice with inclusion of two technical replicates per DNA sample. A TaqMan^®^ Copy Number Reference Assay (Thermo Fisher Scientific, Waltham, Massachusetts, USA) for the transferrin receptor gene (*Tfrc*) was used as a reference with an expected copy number of two. Negative controls lacking DNA template were included for each gene assay, including the reference gene.

Prior to ddPCR, DNA samples were extracted using the Wizard^®^ Genomic DNA Purification Kit (Promega, Madison, Wisconsin, USA), assessed for quantity using a NanoDrop 2000c spectrophotometer (Thermo Fisher Scientific, Waltham, Massachusetts, USA) and diluted to approximately 8 ng/μl. The DNA was then fragmented by centrifuging 140 μl of DNA sample at 16,000xg for 3 min in a QIAshredder column (Qiagen, Venlo, Limburg, Netherlands) to prohibit inaccuracies in copy number detection due to tandem duplications not efficiently sorted in the ddPCR assay [122]. In C57Bl/6 J mice, DNA was extracted from tail samples, with the exception of C57BL/6 J mouse 2 where ear clip tissue was used. DNA was extracted from cerebella for DBA/2 J mice and tail samples for CBA/CaJ mice.

Each 20 μl PCR reaction contained 8 μl of DNA template (~4 ng/μl), 10 μl of the ddPCR™ Supermix for Probes (Bio-Rad, Hercules, California, USA), 1 μl of the FAM™ dye-labelled TaqMan^®^ assay for the gene target of interest, 1 μl of the VIC^®^ dye-labelled TaqMan^®^ reference assay. Droplets were generated by a QX200™ droplet generator (Bio-Rad, Hercules, California, USA). A C1000 Touch™ thermal cycler (Bio-Rad, Hercules, California, USA) was used to run PCR using the following program: 1 cycle at 95 °C for 10 min, 45 cycles of denaturation at 95 °C for 30 s, annealing and extension at 60 °C for 1 min and enzyme deactivation at 98 °C for 10 min.

Droplets were read using a QX200™ droplet reader and analyzed with QuantaSoft™ software (Version 1.7.4.0917; Bio-Rad, Hercules, California, USA).

### Availability of supporting data

The data sets supporting the results of this article are included within the article (and its additional files).

## Additional files

Additional file 1:
**Supplementary Tables and Figures.** X chromosome CNV calls in male and female mice by mouse classification and copy number state. Detailed concordance data with the Mouse Genomes Project and other published study data. Selected highly conserved mammalian genes. Summary of the predicted and experimental ddPCR CN states for nine genic CNVRs in three classical inbred mouse strains and ddPCR confirmation. Number of unique or recurrent^a^ CNV calls on the X chromosome by mouse classification. Probe filtering criteria for SNP, Exon 1 and Exon 2 probes. Details of copy number events on chromosomes 1, 11 and 17. Enrichment and depletion of genomic features in CNV calls, as well as in regions flanking CNV call breakpoints, by size and by strain classification. Principle components analysis for all MGI priority and sequenced strains. Probe density of probe sets used in this study. Additional file 2:
**List of probes used for this analysis.** Probe set identifiers of all probes used in this analysis, one ID per line. Additional file 3:
**CNV calls.** Calls identified in this study for the autosomes for samples passing quality control criteria. Additional file 4:
**Summary of CNV results by sample.** Summary of CNV results for each mouse. Additional file 5:
**Significant results of DAVID analysis.** GO terms associated with CNV results by mouse classification and CNV state. Additional file 6:
**IPA core analysis of genes overlapping CNV regions.** Diseases and functions associated with CNV results by mouse classification and CNV state. Additional file 7:
**List of probes recommended for CNV analysis on MDGA.** Probe set identifiers of all probes recommended for future CNV analysis using the MDGA, one ID per line.

## Abbreviations

aCGH: array comparative genomic hybridization
CN: Copy number
CNA: Copy number alteration
CNV: Copy number variant
CNVR: Copy number variable region
ddPCR: droplet digital PCR
GO: Gene ontology
GRIP: Gene retrocopy insertion polymorphism
IGP: Invariant genomic probe
IPA: Ingenuity Pathway Analysis
LINE: Long interspersed nuclear element
LTR: Long terminal repeat
MDGA: Mouse Diversity Genotyping Array
NGS: Next-generation sequencing
SINE: Short interspersed nuclear element
SNP: Single nucleotide polymorphism
SV: Structural variant
VINO: Variable intensity oligonucleotide
