# Supplementary material for: Genomic copy number variation in Mus musculus
Source: BMC Genomics. 2015 Jul 4;16(1):497. doi: 10.1186/s12864-015-1713-z (PMC4490682; doi:10.1186/s12864-015-1713-z)
Supplement: Additional file 1: — Supplementary Tables and Figures. X chromosome CNV calls in male and female mice by mouse classification and copy number state. Detailed concordance data with the Mouse Genomes Project and other published study data. Selected highly conserved mammalian genes. Summary of the predicted and experimental ddPCR CN states for nine genic CNVRs in three classical inbred mouse strains and ddPCR confirmation. Number of unique or recurrenta CNV calls on the X chromosome by mouse classification. Probe filtering criteria for SNP, Exon 1 and Exon 2 probes. Details of copy number events on chromosomes 1, 11 and 17. Enrichment and depletion of genomic features in CNV calls, as well as in regions flanking CNV call breakpoints, by size and by strain classification. Principle components analysis for all MGI priority and sequenced strains. Probe density of probe sets used in this study. [file 12864_2015_1713_MOESM1_ESM.doc]

## Table S1- X chromosome CNV calls in male mice by mouse classification and copy number state.

| **Mouse classification** | **Number of samples** | **Number of**  **CNV calls** | | **Copy number^a^** | | | | | **Del/**  **amp^b^** |
| --- | --- | --- | --- | --- | --- | --- | --- | --- | --- |
|  |  |  |  | **0** | | **2+** | |  |  |
| All | 279 | 1145 | (4.10) | 433 | (1.55) | 712 | (2.55) |  | 0.61 |
| Classical | 106 | 366 | (3.17) | 73 | (3.45) | 293 | (2.76) |  | 0.25 |
| Wild Derived | 52 | 285 | (5.48) | 165 | (3.17) | 120 | (2.31) |  | 1.35 |
| Wild Caught | 9 | 62 | (6.89) | 44 | (4.89) | 18 | (2) |  | 2.45 |
| C57BL/6J | 5 | 13 | (2.6) | 0 | (0) | 13 | (2.6) |  | 0 |
| C57BL/6NJ | 3 | 6 | (2) | 0 | (0) | 6 | (6) |  | 0 |

The values in parentheses are normalized by sample counts.

^a^ Copy number zero is a deletion, and copy number of 2+ is an amplification in males.

^b^ Deletion/Amplification is the total number of deletions (0 copy-state call counts) divided the number of amplifications (2+ copy-state call counts)

Table S2 - X chromosome CNV calls in female mice by mouse classification and copy number state.

| **Mouse classification** | **Number of samples** | **Number of**  **CNV calls** | | **Copy number^a^** | | | | | | **Del/**  **amp^b^** |
| --- | --- | --- | --- | --- | --- | --- | --- | --- | --- | --- |
|  |  |  |  | **0** | | **1** | | **3+** | |  |
| All | 55 | 73 | (1.33) | 7 | (0.13) | 30 | (0.55) | 36 | (0.65) | 1.02 |
| Classical | 8 | 8 | (1) | 0 | (0) | 1 | (0.13) | 7 | (0.87) | 0.14 |
| Wild Derived | 0 | - | - | - | - | - | - | - | - | - |
| Wild Caught | 10 | 27 | (2.7) | 5 | (0.5) | 10 | (1) | 12 | (1.2) | 1.25 |
| C57BL/6J | 3 | 6 | (2) | 0 | (0) | 0 | (0) | 6 | (2) | 0 |
| C57BL/6NJ | 3 | 1 | (0.33) | 0 | (0) | 0 | (0) | 1 | (0.33) | 0 |

The values in parentheses are normalized by sample counts.

^a^ Copy number zero or one is a deletion, and copy number of 3+ is an amplification in females.

^b^ Deletion/Amplification is the total number of deletions (0 and 1 copy-state call counts) divided the number of amplifications (3+ copy-state call counts).

## Table S3 – Concordance with Mouse Genomes Project^a^ structural variants by strain

| **Strain** | **CEL file ID** | **Total CNV calls** | **Calls overlapping matched strain SVs^b^** | | **Concordance rate^c^** | |
| --- | --- | --- | --- | --- | --- | --- |
| 129S1SvlmJ | SNP_mDIV_A1-1_081308 | 21 | 8 | ( 11 ) | 38.1 | ( 52.4 ) |
| A/J | SNP_mDIV_A2-2_081308 | 33 | 9 | ( 18 ) | 27.3 | ( 54.5 ) |
| AKR/J | SNP_mDIV_A3-3_081308 | 28 | 13 | ( 19 ) | 46.4 | ( 67.9 ) |
| BALB/cJ | SNP_mDIV_D5-253_111308 | 44 | 10 | ( 17 ) | 22.7 | ( 38.6 ) |
| C3H/HeJ | SNP_mDIV_A6-6_081308 | 21 | 6 | ( 8 ) | 28.6 | ( 38.1 ) |
| C57BL/6NJ | SNP_mDIV_A7-SNP08_003_103008 | 12 | 1 | ( 1 ) | 8.3 | ( 8.3 ) |
| C57BL/6NJ | SNP_mDIV_A8-SNP08_003_103008 | 9 | 1 | ( 1 ) | 11.1 | ( 11.1 ) |
| C57BL/6NJ | SNP_mDIV_A9-SNP08_003_103008 | 5 | 0 | ( 0 ) | 0.0 | ( 0 ) |
| C57BL/6NJ | SNP_mDIV_A10-SNP08_004_103008 | 7 | 1 | ( 1 ) | 14.3 | ( 14.3 ) |
| C57BL/6NJ | SNP_mDIV_A11-SNP08_004_103008 | 4 | 1 | ( 1 ) | 25.0 | ( 25.0 ) |
| C57BL/6NJ | SNP_mDIV_B1-SNP08_004_103008_4 | 9 | 1 | ( 1 ) | 11.1 | ( 11.1 ) |
| CBA/J | SNP_mDIV_D8-256_111308 | 22 | 12 | ( 15 ) | 54.5 | ( 68.2 ) |
| DBA/2J | SNP_mDIV_B8-19_081308 | 25 | 10 | ( 16 ) | 40.0 | ( 64.0 ) |
| FVB/NJ | SNP_mDIV_B10-21_081308 | 28 | 11 | ( 20 ) | 39.3 | ( 71.4 ) |
| LP/J | SNP_mDIV_C4-93_090908 | 30 | 10 | ( 19 ) | 33.3 | ( 63.3 ) |
| NOD/ShiLtJ | SNP_mDIV_C6-30_081308 | 17 | 7 | ( 12 ) | 41.2 | ( 70.6 ) |
| NOD/ShiLtJ | SNP_mDIV_C9-404_012709 | 23 | 8 | ( 14 ) | 34.8 | ( 60.9 ) |
| NZO/HiLtJ | SNP_mDIV_C7-31_081308 | 21 | 16 | ( 16 ) | 76.2 | ( 76.2 ) |
| WSB/EiJ | SNP_mDIV_D4-39_081308 | 38 | 17 | ( 24 ) | 44.7 | ( 63.2 ) |

^a^. Wellcome Trust Sanger Institute Mouse Genomes Project Structural Variants Release 1302 [17]

^b^. Overlapping regions are calculated for autosomes only. The number of events having at least 20% reciprocal overlap is listed, followed by the number overlapping by at least 1 bp in brackets.

^c^. Concordance rate is the number of overlapping calls as a percentage of the Total CNV calls for 20% reciprocal overlapping calls followed 1bp overlapping in brackets.

## Table S4 – Call state and recurrence concordance with Mouse Genomes Project^a^ Structural Variants by strain

|  |  | CNV call counts at copy-states 0-4 | | | | | | | | Recurrent CNV call counts | | Sanger Call Type for Overlapping^d^ | | | |
| --- | --- | --- | --- | --- | --- | --- | --- | --- | --- | --- | --- | --- | --- | --- | --- |
|  |  | Not^b^ | | | | Over^c^ | | | | Not^b^ | Over^c^ |  |  |  |  |
| Strain | CEL ID | 0 | 1 | 3 | 4 | 0 | 1 | 3 | 4 |  |  | H8 | H10 | H11 | Del |
| 129S1SvlmJ | A1-1_081308 | 1 | 2 | 6 | 4 | 0 | 3 | 4 | 1 | 12 | 8 | 3 | 3 | 1 | 2 |
| A/J | A2-2_081308 | 1 | 9 | 11 | 3 | 2 | 1 | 3 | 3 | 17 | 9 | 5 | 1 | 0 | 4 |
| AKR/J | A3-3_081308 | 0 | 4 | 1 | 10 | 4 | 4 | 2 | 3 | 11 | 10 | 4 | 2 | 0 | 8 |
| BALB/cJ | D5-253_111308 | 1 | 8 | 21 | 4 | 1 | 2 | 4 | 3 | 18 | 7 | 3 | 4 | 0 | 3 |
| C3H/HeJ | A6-6_081308 | 0 | 5 | 3 | 7 | 2 | 1 | 1 | 2 | 13 | 6 | 2 | 1 | 0 | 4 |
| C57BL/6NJ | A10-SNP08_004_103008 | 0 | 3 | 1 | 2 | 1 | 0 | 0 | 0 | 6 | 1 | 0 | 0 | 0 | 1 |
| C57BL/6NJ | A11-SNP08_004_103008 | 0 | 1 | 0 | 2 | 1 | 0 | 0 | 0 | 3 | 1 | 0 | 0 | 0 | 1 |
| C57BL/6NJ | A7-SNP08_003_103008 | 0 | 7 | 0 | 4 | 1 | 0 | 0 | 0 | 11 | 1 | 0 | 0 | 0 | 1 |
| C57BL/6NJ | A8-SNP08_003_103008 | 0 | 3 | 3 | 2 | 1 | 0 | 0 | 0 | 8 | 1 | 0 | 0 | 0 | 1 |
| C57BL/6NJ | A9-SNP08_003_103008 | 0 | 3 | 0 | 2 | 0 | 0 | 0 | 0 | 5 | 0 | 0 | 0 | 0 | 0 |
| C57BL/6NJ | B1-SNP08_004_103008_4 | 0 | 6 | 0 | 2 | 1 | 0 | 0 | 0 | 8 | 1 | 0 | 0 | 0 | 1 |
| CBA/J | D8-256_111308 | 2 | 6 | 1 | 1 | 3 | 3 | 4 | 2 | 9 | 10 | 2 | 5 | 1 | 9 |
| DBA/2J | B8-19_081308 | 2 | 6 | 3 | 4 | 1 | 3 | 5 | 1 | 14 | 9 | 3 | 3 | 0 | 5 |
| FVB/NJ | B10-21_081308 | 1 | 6 | 5 | 5 | 3 | 3 | 3 | 2 | 17 | 11 | 3 | 4 | 1 | 5 |
| LP/J | C4-93_090908 | 2 | 6 | 6 | 6 | 1 | 2 | 5 | 2 | 17 | 9 | 4 | 4 | 0 | 2 |
| NOD/ShiLtJ | C6-30_081308 | 0 | 2 | 2 | 6 | 2 | 1 | 3 | 1 | 8 | 7 | 1 | 4 | 0 | 2 |
| NOD/ShiLtJ | C9-404_012709 | 0 | 4 | 5 | 6 | 2 | 1 | 3 | 2 | 13 | 8 | 2 | 4 | 0 | 2 |
| NZO/HiLtJ | C7-31_081308 | 0 | 2 | 0 | 3 | 7 | 1 | 2 | 6 | 4 | 15 | 3 | 5 | 0 | 9 |
| WSB/EiJ | D4-39_081308 | 7 | 7 | 4 | 3 | 12 | 0 | 3 | 2 | 18 | 16 | 2 | 3 | 0 | 14 |

^a^. Wellcome Trust Sanger Institute Mouse Genomes Project Structural Variants Release 1302 [17]

^b^. Not – refers to count of calls not overlapping with a call from the Mouse Genomes Project

^c^. Over – refers to count of calls overlapping with a call from the Mouse Genomes Project

^d^. Call types as specified by [17]

## Table S5 – Number of CNV calls overlapping previously reported variable regions

| **Report^a^** | **Number of**  **reported regions^b^** | **Reported regions overlapping**  **regions from this study^c^** | | **Regions from this study overlapping**  **reported regions^c^** | |
| --- | --- | --- | --- | --- | --- |
| Simon 2013 | 43 | 4 | (6) | 13 | (17) |
| Nellåker 2012 | 110930 | 91 | (7527) | 244 | (5511) |
| Yalcin 2012 | 1453 | 73 | (559) | 698 | (968) |
| Wong 2012 | 30042 | 107 | (2284) | 1084 | (3612) |
| Quinlan 2012 | 7371 | 173 | (708) | 1610 | (2733) |
| Keane 2011 | 711915 | 1726 | (48647) | 3778 | (7900) |
| **Total NGS** |  |  | | **4105** | **(8047)** |
| Yang 2011 | 178 | 191 | (354) | 128 | (142) |
| **Total MDGA** |  |  |  | **128** | **(142)** |
| Bryk 2014 | 1868 | 59 | (181) | 543 | (730) |
| Agam 2010 | 1976 | 248 | (426) | 1378 | (2298) |
| Henrichsen 2009 | 7090 | 3985 | (6862) | 1582 | (2441) |
| Cahan 2009 | 1333 | 779 | (1250) | 2460 | (3165) |
| She 2008 | 3118 | 153 | (442) | 629 | (976) |
| Egan 2007 | 38 | 3 | (15) | 30 | (61) |
| Graubert 2007 | 297 | 130 | (158) | 1010 | (1212) |
| Cutler 2007 | 2116 | 902 | (1079) | 2805 | (3416) |
| **Total aCGH** |  |  | | **4250** | **(5573)** |
| **Regions from this study** | **9634** | **---** | | **5346** | **(8455)** |

^a^ Reports are divided by a horizontal lines to indicate detection by NGS (top section), MDGA, or aCGH (bottom section).

^b^ The number of previously reported regions includes all regions reported, sets are not reduced to have only unique reported regions and can also include the X and Y chromosomes.

^c^ Overlapping regions are calculated for autosomes only. The number of events having at least 20% reciprocal overlap is listed, followed by the number overlapping by at least 1 bp in brackets.

## Table S6 - Selected highly conserved mammalian genes

| **Gene Symbol^a^** | **Ensemble Gene ID** |
| --- | --- |
| *Ttn* | ENSMUSG00000051747 |
| *Cnr1* | ENSMUSG00000044288 |
| *Bche* | ENSMUSG00000027792 |
| *S1pr1* (*EDG1*) | ENSMUSG00000045092 |
| *Rag1* | ENSMUSG00000061311 |
| *Rag2* | ENSMUSG00000032864 |
| *Atp7a* | ENSMUSG00000033792 |
| *Tyr* (*TYR1*) | ENSMUSG00000004651 |
| *Adora3* | ENSMUSG00000074344 |
| *Bdnf* | ENSMUSG00000048482 |
| *Adrb2* | ENSMUSG00000045730 |
| *Pnoc* | ENSMUSG00000045731 |
| *Adra2b* (*A2AB*) | ENSMUSG00000058620 |
| *Brca1* | ENSMUSG00000017146 |
| *Brca2* | ENSMUSG00000041147 |
| *Dmp1* | ENSMUSG00000029307 |
| *Ghr* | ENSMUSG00000055737 |
| *Vwf* | ENSMUSG00000001930 |
| *Enam* | ENSMUSG00000029286 |
| *Apob* | ENSMUSG00000020609 |
| *Rbp3* (*IRBP*) | ENSMUSG00000041534 |
| *App* | ENSMUSG00000022892 |
| *Bmi1* | ENSMUSG00000026739 |
| *Crem* | ENSMUSG00000063889 |
| *Fbn1* | ENSMUSG00000027204 |
| *Plcb4* | ENSMUSG00000039943 |

Genes identified as being highly conserved in mammals, and unlikely to be found having copy number variation, in particular, deletions.

^a^Gene names are listed for mouse (Mouse Genome Informatics symbol), the alternate symbol identified in [49] is listed in brackets when different.

## Table S7 - Summary of the predicted and experimental ddPCR CN states for nine genic CNVRs in three classical inbred mouse strains

| **CNVR gene target** | **TaqMan Assay ID** | **CNVR location^a^** | **CNVR size (bp)** | **Number of SNP probes in CNVR** | **Number of IGPs in CNVR** | **Predicted CN State Summary** | | | **ddPCR CN State Summary** | | |
| --- | --- | --- | --- | --- | --- | --- | --- | --- | --- | --- | --- |
|  |  |  |  |  |  | **C57BL/6J (n = 8)** | **CBA/CaJ (n = 1)** | **DBA/2J (n = 1)** | **C57BL/6J mice** | **CBA/CaJ mice** | **DBA/2J mice** |
| ***B4galt3 ^b^*** | Mm00735212_cn | chr1:173206026-173208119 | 2093 | 2 | 18 | 2 (5), 3 (3)^c^ | 2 | 2 | 2 | 2 | 2 |
| ***Itln1*** | Mm00534147_cn | chr1:173391344-173508077 | 116733 | 12 | 43 | 1 (8) | 2 | 2 | 2 | 2 | 2 |
| ***Nlrp1b ^b^*** | Mm00635379_cn | chr11:71014664-71099017 | 84353 | 3 | 29 | 2 (1), 3 (2), 4 (5) | 2 | 2 | 2 | 2 | 2 |
| ***Hdhd3*** | Mm00553493_cn | chr4:62157378-62185081 | 27703 | 3 | 33 | 2 (8) | 4 | 3 | 2 | 6 | 6 |
| ***Skint3*** | Mm00735949_cn | chr4:111745396-112064463 | 319067 | 0 | 61 | 4 (2,6) | 2 | 2 | 2 | 0 | 0 |
| ***Trim30e-ps1*** | Mm00657977_cn | chr7:111681502-111683670 | 2168 | 0 | 7 | 2 (5), 4 (3) | 2 | 2 | 2 | 0 | 0 |
| ***Ide*** | Mm00496897_cn | chr19:37317974-37451133 | 133159 | 31 | 54 | 2 (2), 3 (5,*1*) | 2 | 2 | 2 | 2 | 2 |
| ***Fgfbp3*** | Mm00630217_cn | chr19:36990335-37045706 | 55371 | 12 | 38 | 2 (5), 3 (3) | 2 | 2 | 2 and 3 | 2 | 2 |
| ***Glo1*** | Mm00735212_cn | chr17:30593663-31058945 | 465282 | 61 | 273 | 1(6), 2 (2) | 3 | 3 | 2 | 4 | 4 |

a The CNVR start and end positions are determined by the earliest start position and latest end position in a group of overlapping CNVs for CNVs detected in C57BL/6J, CBA/CaJ and DBA/2J mice.

b These genes partially overlap the CNVR.

c The number of mice where the observed CNV only partially overlapped the gene of interest is underlined.

## Table S8 - ddPCR confirmation of CN state in 14 mice of three different inbred strains for nine select genic CNVRs detected using the Mouse Diversity Genotyping Array.

| **Gene Assay** | **Predicted CN state** | | | **ddPCR CN state** | | | | | | | | | | | | | |
| --- | --- | --- | --- | --- | --- | --- | --- | --- | --- | --- | --- | --- | --- | --- | --- | --- | --- |
|  | **C57BL/6J (n = 8)** | **CBA/CaJ (n = 1)** | **DBA/2J (n = 1)** | **C57B/6J mouse** | | | | | **CBA/CaJ mouse** | | | | | **DBA/2J mouse** | | | |
|  |  |  |  | **1** | **2** | **3** | **4** | **5** | **1** | **2** | **3** | **4** | **5** | **1** | **2** | **3** | **4** |
| ***B4galt3*** | 2 (5), 3 (3)^d^ | 2 | 2 | 1.99 | 1.80 | 2.01 | 1.90 | 1.94 | 2.04 | 2.09 | 2.02 | 1.97 | 1.89 | 2.01 | 1.94 | 1.94 | 1.97 |
|  |  |  |  | 1.87 | 1.87 | 1.87 | 1.99 | 2.00 | 2.02 | 2.17 | 2.00 | 1.85 | 1.96 | 1.87 | 1.90 | 2.02 | 2.03 |
| ***Itln1*^a,b^** | 1 (8) | 2 | 2 | 1.96 | 1.89 | 1.95 | 1.95 | 2.06 | 1.94 | 1.92 | 1.98 | 1.93 | 1.92 | 1.95 | 1.89 | 2.01 | 1.94 |
|  |  |  |  | 1.90 | 1.90 | 2.03 | 2.02 | 1.97 | 1.90 | 1.93 | 1.96 | 1.88 | 2.06 | 2.01 | 1.94 | 1.99 | 1.92 |
| ***Nlrp1b*** | 2 (1), 3 (2), 4 (5) | 2 | 2 | 2.09 | 2.01 | 2.01 | 1.99 | 1.86 | 1.98 | 1.96 | 1.95 | 1.92 | 1.99 | 2.09 | 2.11 | 2.04 | 2.06 |
|  |  |  |  | 2.09 | 1.97 | 1.89 | 1.99 | 1.97 | 1.99 | 2.01 | 2.04 | 1.99 | 2.02 | 1.96 | 2.04 | 1.98 | 1.99 |
| ***Hdhd3^a,c^*** | 2 (8) | 4 | 3 | 1.93 | 1.78 | 1.98 | 1.98 | 1.96 | 5.85 | 5.87 | 5.93 | 6.02 | 5.86 | 5.66 | 5.90 | 5.90 | 5.70 |
|  |  |  |  | 1.96 | 1.71 | 1.95 | 1.98 | 1.96 | 5.80 | 5.80 | 6.01 | 5.78 | 5.81 | 5.69 | 5.71 | 5.89 | 5.90 |
| ***Skint3*** | 4 (2,6)^e^ | 2 | 2 | 2.09 | 2.19 | 1.91 | 2.08 | 2.09 | 0.00 | 0.00 | 0.00 | 0.00 | 0.00 | 0.00 | 0.00 | 0.00 | 0.00 |
|  |  |  |  | 2.02 | 2.04 | 2.02 | 1.98 | 1.99 | 0.00 | 0.00 | 0.00 | 0.00 | 0.00 | 0.00 | 0.00 | 0.00 | 0.00 |
| ***Trim30e-ps1*** | 2 (5), 4 (3) | 2 | 2 | 2.07 | 1.88 | 1.98 | 2.10 | 1.94 | 0.00 | 0.00 | 0.00 | 0.00 | 0.00 | 0.00 | 0.00 | 0.00 | 0.00 |
|  |  |  |  | 2.00 | 1.91 | 1.94 | 2.01 | 1.99 | 0.00 | 0.00 | 0.00 | 0.00 | 0.00 | 0.00 | 0.00 | 0.00 | 0.00 |
| ***Ide*** | 2 (2), 3 (5,*1*) | 2 | 2 | 1.89 | 1.86 | 1.92 | 1.88 | 1.87 | 1.91 | 1.90 | 1.94 | 1.89 | 1.94 | 1.97 | 1.94 | 1.86 | 1.99 |
|  |  |  |  | 1.94 | 1.89 | 1.93 | 1.92 | 1.89 | 1.94 | 1.91 | 2.01 | 1.94 | 1.90 | 1.99 | 1.94 | 1.96 | 1.97 |
| ***Fgfbp3^a^*** | 2 (5), 3 (3) | 2 | 2 | 1.97 | 2.92 | 2.06 | 2.07 | 2.12 | 2.02 | 2.11 | 2.00 | 1.99 | 2.11 | 2.11 | 2.09 | 2.00 | 2.05 |
|  |  |  |  | 2.04 | 2.89 | 2.03 | 2.04 | 2.04 | 2.05 | 2.03 | 2.15 | 2.00 | 2.05 | 1.99 | 2.02 | 1.97 | 2.00 |
| ***Glo1^a^*** | 1(6), 2 (2) | 3 | 3 | 1.89 | 2.04 | 2.05 | 1.94 | 1.94 | 3.94 | 3.88 | 3.84 | 3.97 | 3.99 | 3.86 | 4.12 | 3.87 | 3.90 |
|  |  |  |  | 2.01 | 1.97 | 1.97 | 2.01 | 1.99 | 3.93 | 3.94 | 3.99 | 4.00 | 3.99 | 3.96 | 4.10 | 3.94 | 3.74 |

a The gene is entirely within the CNV observed in each mouse.

b All DBA/2J and CBA/CaJ samples had multiple droplet populations between the lowest amplitude negative droplet population and the highest amplitude Itln1 postive droplet population. This may be caused by genetic variation in the probe and primer regions that reduces amplification efficiency.

c For C57BL/6J mouse 2, the CN is likely 2 but ddPCR values were lower than expected for a CN of 2, possibly because the duplicate regions are very close in proximity and further fragmentation of the sample is required to accurately detect the CN state.

d The number of mice where the observed CNV only partially overlapped the gene of interest is underlined.

eFor the case of 6 C57Bl/6J mice with only a partial overlap of the CNVR and the Skint3 gene, 2 is also a potential CN state.

## Table S9 - Number of unique or recurrent^a^ CNV calls on the X chromosome by mouse classification

| **Mouse classification** | **Number of samples** | **CNV occurrence^a^** | | | | **Unique/**  **recurrent** |
| --- | --- | --- | --- | --- | --- | --- |
|  |  | **Unique** | | **Recurrent^b^** | |  |
| All | 334 | 213 | (0.64) | 1005 | (3.01) | 0.21 |
| Classical | 114 | 24 | (0.21) | 350 | (3.07) | 0.07 |
| Wild derived | 52 | 61 | (1.17) | 224 | (4.31) | 0.27 |
| Wild caught | 19 | 33 | (1.74) | 56 | (2.95) | 0.59 |
| C57BL/6J | 8 | 2 | (0.25) | 17 | (2.13) | 0.12 |
| C57BL/6NJ | 6 | 0 | (0) | 7 | (1.17) | 0.00 |
| Male | 279 | 191 | (0.68) | 954 | (3.42) | 0.20 |
| Female | 55 | 22 | (0.40) | 51 | (0.93) | 0.43 |

^a^ Uniqueness and recurrence are both consistently based on the entire analysis, and are not reevaluated within mouse classification types (classical, wild derived, etc.), i.e. a call being unique in the wild caught group was not found to overlap with any other call in the entire analysis, and is not only unique within the samples classified as wild caught. In brackets, the call count is normalized by sample size.

^b^ A call is considered recurrent if it has 40% reciprocal overlap with any other call

## Figure S1- Probe filtering criteria for SNP, Exon 1 and Exon 2 probes


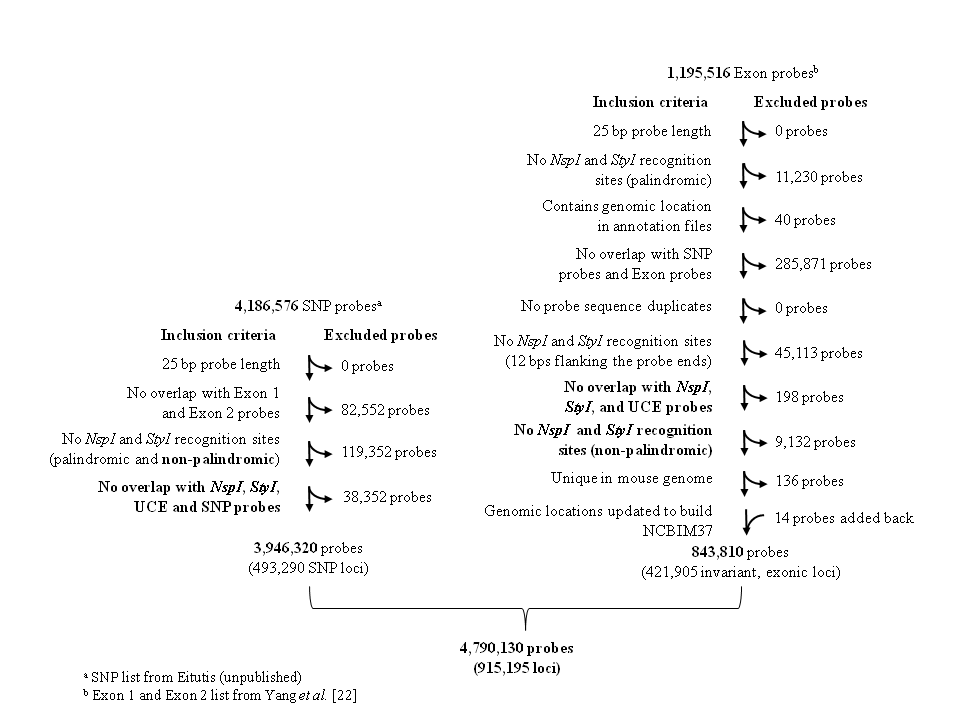


Bolded criteria were used in addition to the other criteria to construct the stringent probe list.

## Figure S2 - Copy number events on Chr17: 30500000-31060200

**
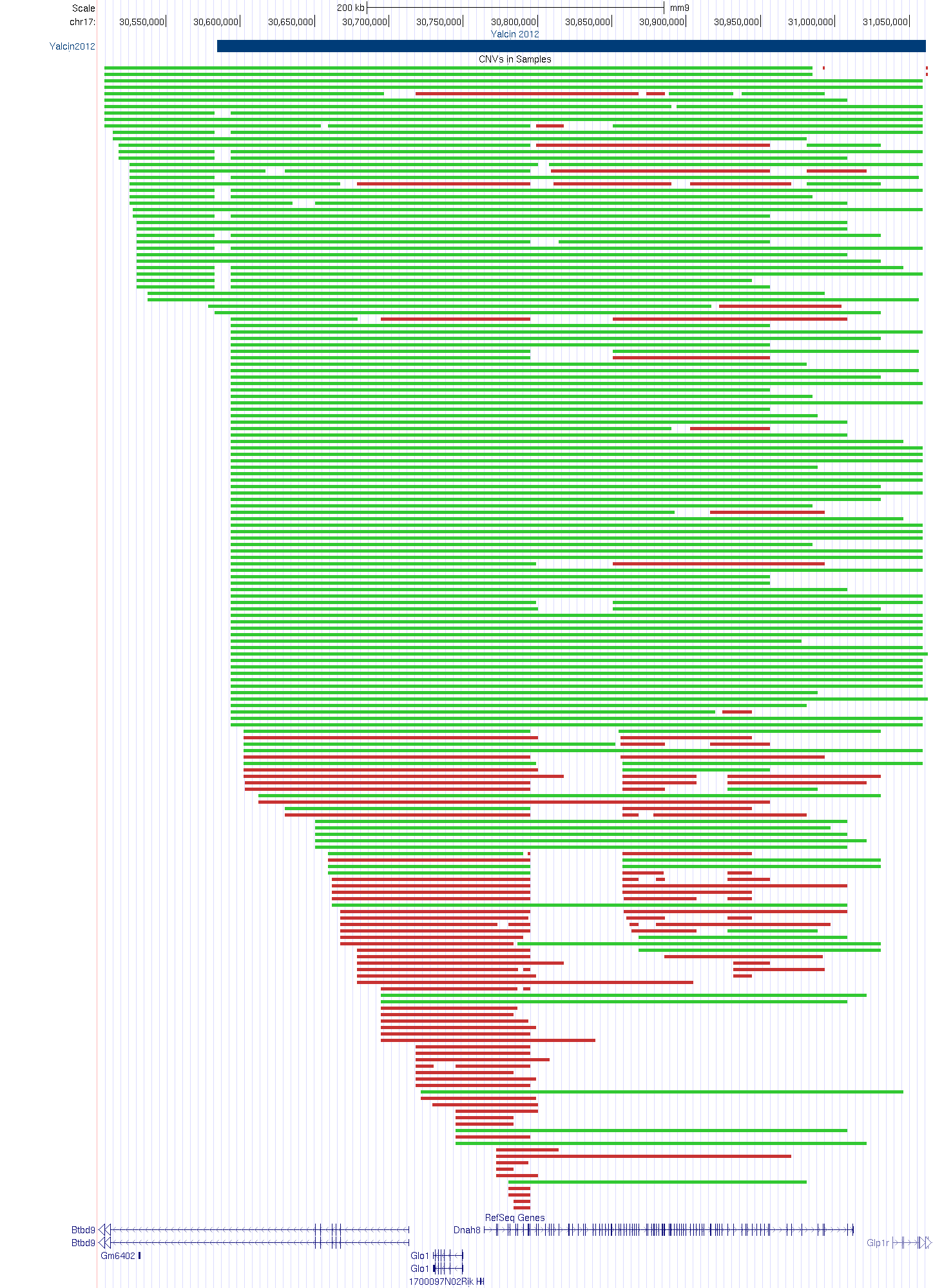
**

Comparison to event reported in Yalcin et al. 2012 [14] (shown at top in dark blue). Copy number amplifications are denoted in green, deletions in red.

## Figure S3 - Copy number events on Chr1: 90100000-90210000

**
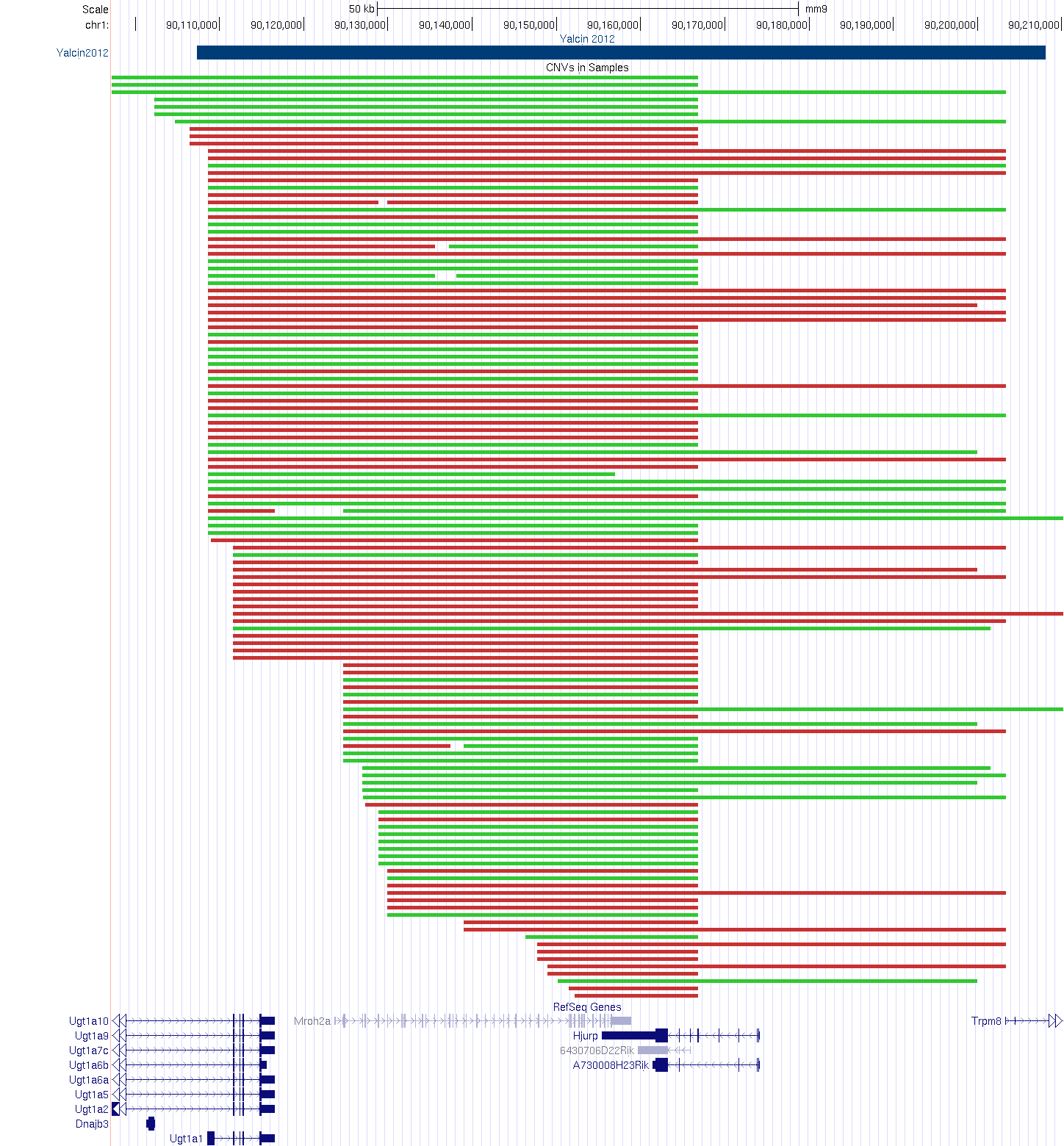
**

Comparison to event reported in Yalcin et al. 2012 [14] (shown at top in dark blue). Copy number amplifications are denoted in green, deletions in red.

## Figure S4 - Copy number events on Chr11: 70900000-71100000


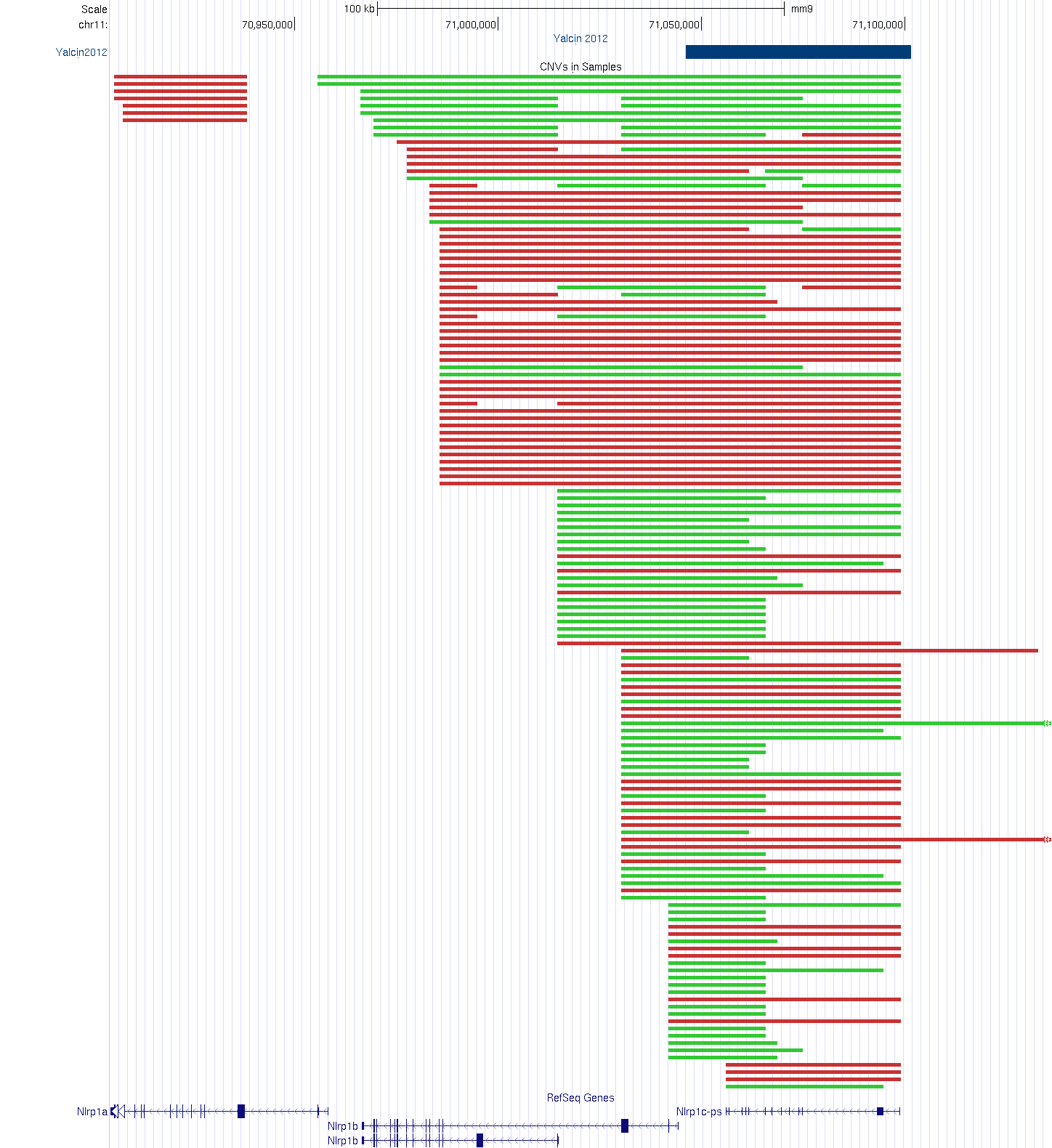


Comparison to event reported in Yalcin et al. 2012 [14] (shown at top in dark blue). Copy number amplifications are denoted in green, deletions in red.

## Figure S5 - Enrichment and depletion of genomic features in CNV calls by size


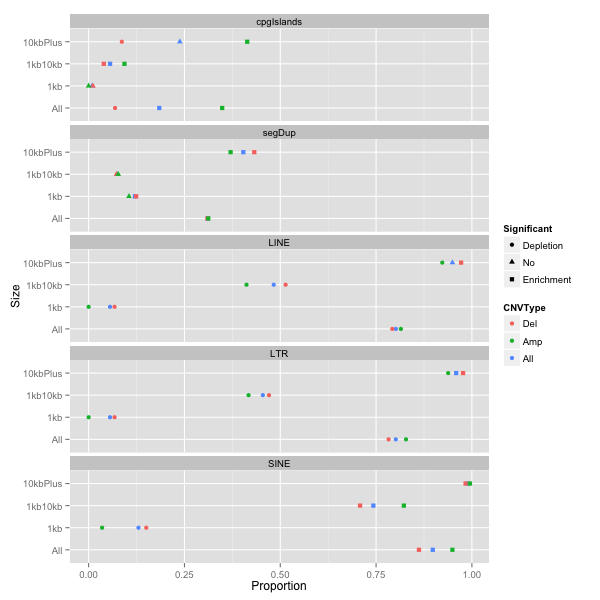


## Figure S6 - Enrichment and depletion of genomic elements in regions flanking CNV call breakpoints by size


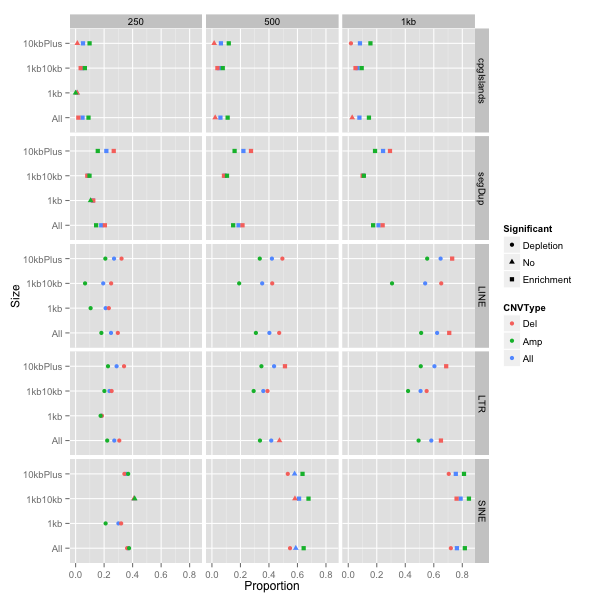


## Figure S7 - Enrichment and depletion of genomic features in CNV calls by strain classification

##
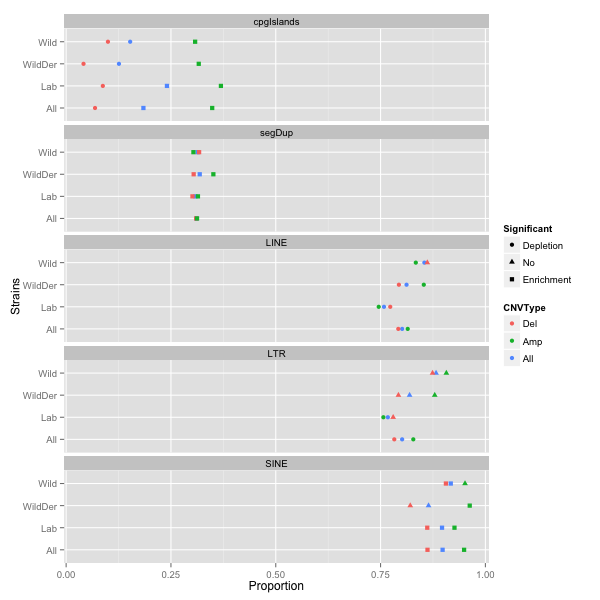


## Figure S8 - Principle components analysis for all MGI priority and sequenced strains

**
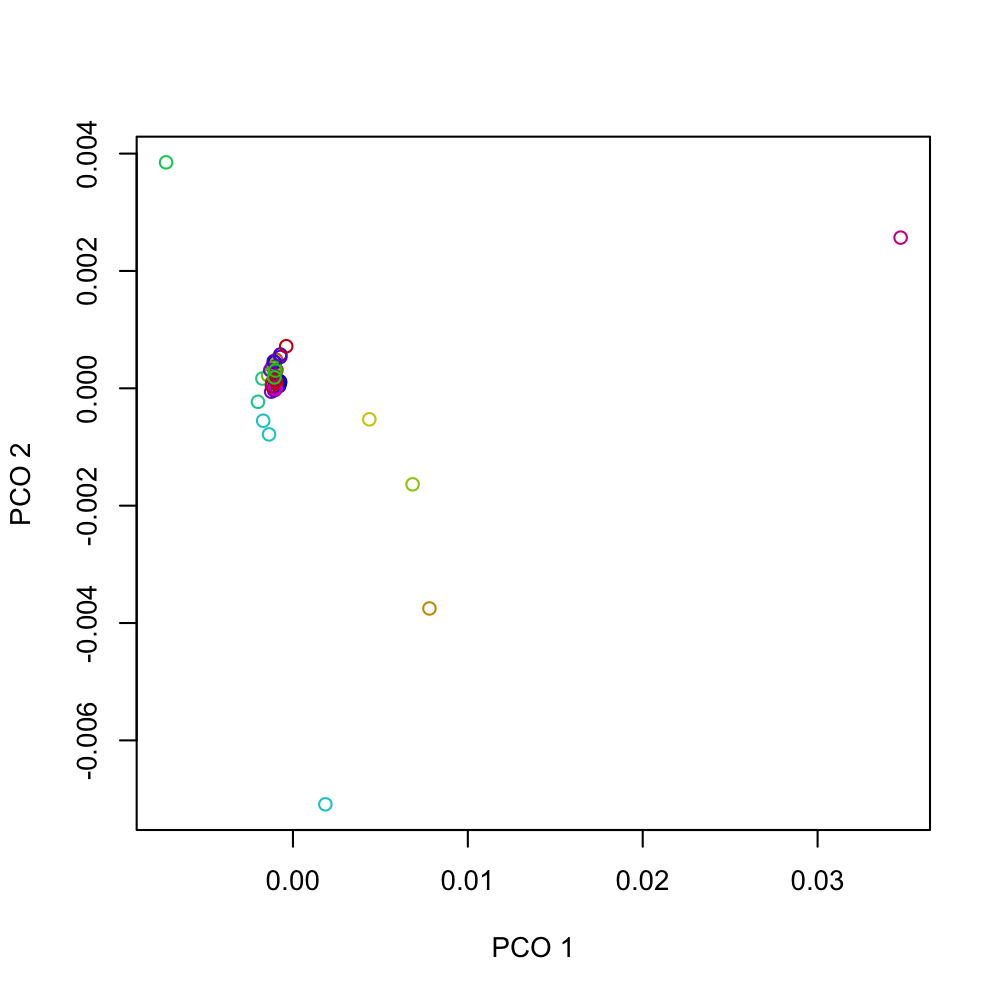
**

## Figure S9 - Probe density of probe sets used in this study

**
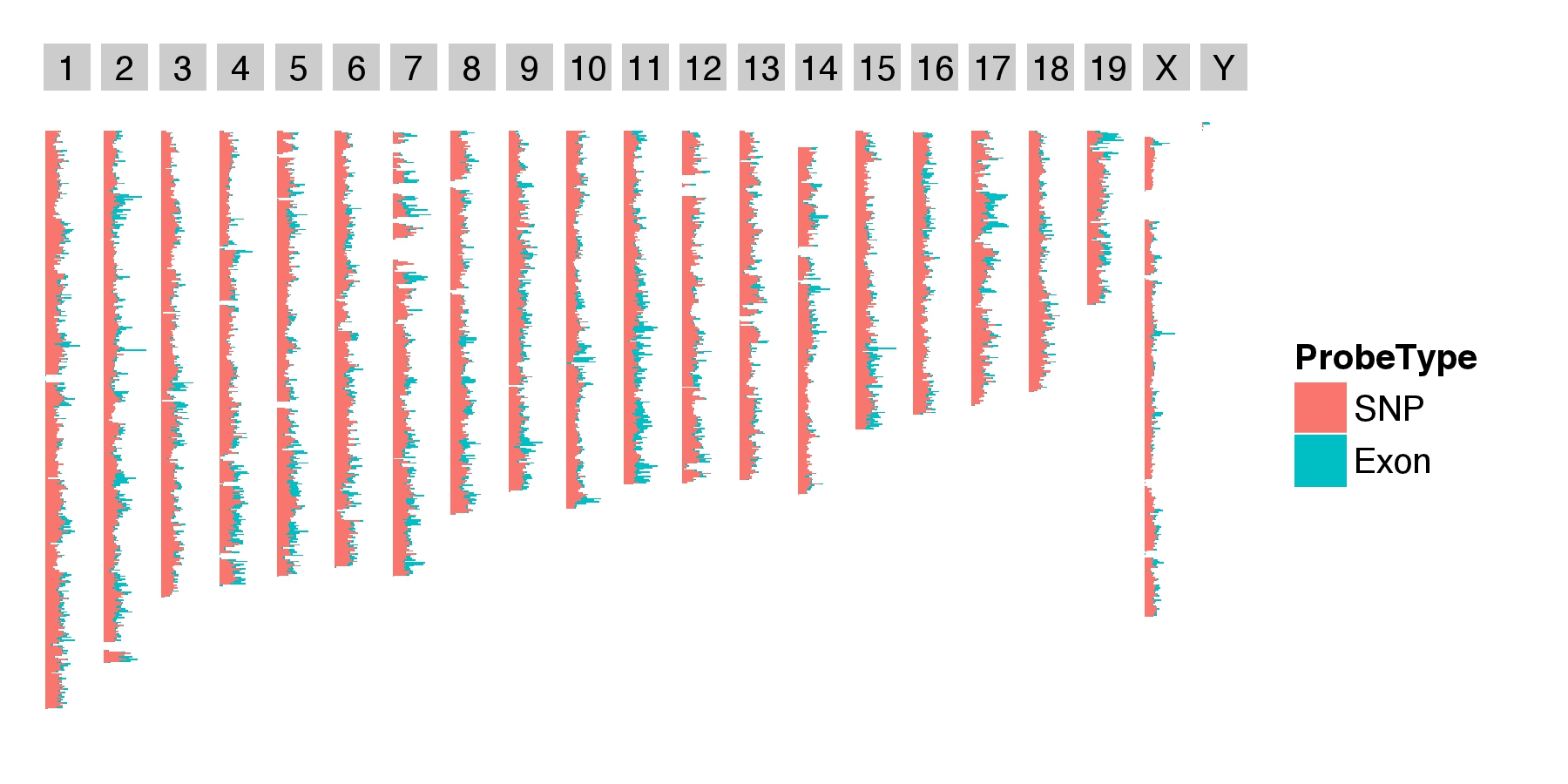
**

Histogram over the genome of probe count by probe type (SNP probe or Exon IGP probe).
