# Supplementary material for: Genomic copy number variation in Mus musculus
Source: BMC Genomics. 2015 Jul 4;16(1):497. doi: 10.1186/s12864-015-1713-z (PMC4490682; doi:10.1186/s12864-015-1713-z)
Supplement: Additional file 5: — Significant results of DAVID analysis. GO terms associated with CNV results by mouse classification and CNV state. [file 12864_2015_1713_MOESM5_ESM.doc]

**Enriched DAVID Gene Ontology (GO) terms for genic CNVs**

| **Mouse Classification** | **CNV State** | **Category^1^** | **Gene Ontology Term** | **Involved genes** | **Involved genes/total genes inputted (%)** | **Fold Enrichment** | **P*_adj_*(Benjamini-Hochberg)^2^** |
| --- | --- | --- | --- | --- | --- | --- | --- |
| Classical | 3+ | BP | GO:0048002~antigen processing and presentation of peptide antigen | 16 | 1.95 | 13.08 | 3.26E-10 |
| Classical | 3+ | BP | GO:0019882~antigen processing and presentation | 22 | 2.68 | 7.23 | 1.24E-09 |
| Classical | 3+ | CC | GO:0042611~MHC protein complex | 17 | 2.07 | 9.19 | 5.60E-09 |
| Classical | 3+ | CC | GO:0000786~nucleosome | 16 | 1.95 | 7.25 | 5.12E-07 |
| Classical | 3+ | BP | GO:0006334~nucleosome assembly | 17 | 2.07 | 6.66 | 1.81E-06 |
| Classical | 3+ | BP | GO:0065004~protein-DNA complex assembly | 17 | 2.07 | 6.40 | 2.02E-06 |
| Classical | 3+ | BP | GO:0034728~nucleosome organization | 17 | 2.07 | 6.40 | 2.02E-06 |
| Classical | 3+ | BP | GO:0031497~chromatin assembly | 17 | 2.07 | 6.48 | 2.06E-06 |
| Classical | 3+ | CC | GO:0032993~protein-DNA complex | 16 | 1.95 | 5.99 | 5.47E-06 |
| Classical | 3+ | BP | GO:0002474~antigen processing and presentation of peptide antigen via MHC class I | 9 | 1.10 | 15.14 | 8.19E-06 |
| Classical | 3+ | BP | GO:0006333~chromatin assembly or disassembly | 19 | 2.32 | 4.99 | 9.24E-06 |
| Classical | 3+ | BP | GO:0002478~antigen processing and presentation of exogenous peptide antigen | 10 | 1.22 | 12.44 | 1.07E-05 |
| Classical | 3+ | BP | GO:0006323~DNA packaging | 18 | 2.20 | 5.10 | 1.29E-05 |
| Classical | 3+ | BP | GO:0065003~macromolecular complex assembly | 34 | 4.15 | 2.88 | 1.60E-05 |
| Classical | 3+ | BP | GO:0002504~antigen processing and presentation of peptide or polysaccharide antigen via MHC class II | 9 | 1.10 | 13.55 | 1.74E-05 |
| Classical | 3+ | BP | GO:0043933~macromolecular complex subunit organization | 35 | 4.27 | 2.73 | 2.99E-05 |
| Classical | 3+ | CC | GO:0042613~MHC class II protein complex | 7 | 0.85 | 19.67 | 3.23E-05 |
| Classical | 3+ | BP | GO:0019884~antigen processing and presentation of exogenous antigen | 10 | 1.22 | 10.22 | 3.59E-05 |
| Classical | 3+ | MF | GO:0004886~retinoid-X receptor activity | 7 | 0.85 | 23.02 | 6.11E-05 |
| Classical | 3+ | BP | GO:0019886~antigen processing and presentation of exogenous peptide antigen via MHC class II | 8 | 0.98 | 14.30 | 6.43E-05 |
| Classical | 3+ | BP | GO:0002495~antigen processing and presentation of peptide antigen via MHC class II | 8 | 0.98 | 14.30 | 6.43E-05 |
| Classical | 3+ | BP | GO:0034622~cellular macromolecular complex assembly | 25 | 3.05 | 3.30 | 6.72E-05 |
| Classical | 3+ | MF | GO:0042809~vitamin D receptor binding | 7 | 0.85 | 20.72 | 7.42E-05 |
| Classical | 3+ | CC | GO:0044427~chromosomal part | 31 | 3.78 | 2.74 | 7.53E-05 |
| Classical | 3+ | MF | GO:0030375~thyroid hormone receptor coactivator activity | 7 | 0.85 | 18.83 | 1.06E-04 |
| Classical | 3+ | MF | GO:0010861~thyroid hormone receptor activator activity | 7 | 0.85 | 18.83 | 1.06E-04 |
| Classical | 3+ | MF | GO:0030546~receptor activator activity | 7 | 0.85 | 17.26 | 1.54E-04 |
| Classical | 3+ | MF | GO:0003708~retinoic acid receptor activity | 7 | 0.85 | 17.26 | 1.54E-04 |
| Classical | 3+ | BP | GO:0034621~cellular macromolecular complex subunit organization | 26 | 3.17 | 3.04 | 1.66E-04 |
| Classical | 3+ | CC | GO:0000785~chromatin | 20 | 2.44 | 3.41 | 3.69E-04 |
| Classical | 3+ | MF | GO:0046966~thyroid hormone receptor binding | 7 | 0.85 | 14.80 | 3.78E-04 |
| Classical | 3+ | BP | GO:0006325~chromatin organization | 29 | 3.54 | 2.63 | 5.65E-04 |
| Classical | 3+ | CC | GO:0042612~MHC class I protein complex | 10 | 1.22 | 6.69 | 5.81E-04 |
| Classical | 3+ | CC | GO:0005694~chromosome | 32 | 3.90 | 2.38 | 6.51E-04 |
| Classical | 3+ | BP | GO:0051276~chromosome organization | 33 | 4.02 | 2.34 | 0.00128402 |
| Classical | 3+ | MF | GO:0042974~retinoic acid receptor binding | 7 | 0.85 | 11.51 | 0.00173474 |
| Classical | 3+ | MF | GO:0030545~receptor regulator activity | 7 | 0.85 | 10.90 | 0.00211124 |
| Classical | 3+ | MF | GO:0030374~ligand-dependent nuclear receptor transcription coactivator activity | 7 | 0.85 | 9.86 | 0.00348611 |
| Wild Caught | 0 | MF | GO:0004984~olfactory receptor activity | 12 | 16.00 | 4.39 | 0.00223459 |
| Wild Caught | 1 | BP | GO:0007606~sensory perception of chemical stimulus | 71 | 26.59 | 5.16 | 1.46E-30 |
| Wild Caught | 1 | BP | GO:0007600~sensory perception | 72 | 26.97 | 4.44 | 1.74E-27 |
| Wild Caught | 1 | BP | GO:0050877~neurological system process | 78 | 29.21 | 4.02 | 2.25E-27 |
| Wild Caught | 1 | BP | GO:0050890~cognition | 72 | 26.97 | 4.21 | 3.88E-26 |
| Wild Caught | 1 | MF | GO:0004984~olfactory receptor activity | 62 | 23.22 | 4.81 | 1.10E-24 |
| Wild Caught | 1 | BP | GO:0007608~sensory perception of smell | 61 | 22.85 | 4.73 | 8.03E-24 |
| Wild Caught | 1 | BP | GO:0007186~G-protein coupled receptor protein signaling pathway | 76 | 28.46 | 3.50 | 4.46E-23 |
| Wild Caught | 1 | BP | GO:0007166~cell surface receptor linked signal transduction | 79 | 29.59 | 2.74 | 1.50E-17 |
| Wild Caught | 1 | CC | GO:0016021~integral to membrane | 101 | 37.83 | 1.46 | 4.06E-05 |
| Wild Caught | 1 | CC | GO:0031224~intrinsic to membrane | 102 | 38.20 | 1.42 | 6.84E-05 |
| Wild Caught | 1 | MF | GO:0016503~pheromone receptor activity | 11 | 4.12 | 7.17 | 3.08E-04 |
| Wild Caught | 3+ | MF | GO:0005550~pheromone binding | 13 | 5.39 | 15.33 | 9.35E-09 |
| Wild Caught | 3+ | MF | GO:0005549~odorant binding | 13 | 5.39 | 14.17 | 1.20E-08 |
| Wild Caught | 3+ | BP | GO:0019236~response to pheromone | 13 | 5.39 | 13.92 | 1.03E-07 |
| Wild Caught | 3+ | MF | GO:0016503~pheromone receptor activity | 13 | 5.39 | 11.13 | 1.36E-07 |
| Wild Caught | 3+ | CC | GO:0031226~intrinsic to plasma membrane | 20 | 8.30 | 4.23 | 1.33E-05 |
| Wild Caught | 3+ | CC | GO:0005887~integral to plasma membrane | 20 | 8.30 | 4.40 | 1.45E-05 |
| Wild Caught | 3+ | MF | GO:0004965~GABA-B receptor activity | 5 | 2.07 | 48.14 | 1.46E-04 |
| Wild Caught | 3+ | BP | GO:0010033~response to organic substance | 17 | 7.05 | 3.75 | 4.22E-03 |

^1^ BP, Biological Process; CC, Cellular Component; MF, Molecular Function

^2^Only GO terms with significant *P*-values (*P_adj_* <0.05) are included
